# Mid-IR standoff measurement of ageing-related spectroscopic changes in bitumen in the 6 µm (1700 cm^−1^) region. Part 2: Instrument development and results

**DOI:** 10.1038/s41598-025-02062-3

**Published:** 2025-07-08

**Authors:** Nicholas M. Davis, Jane Hodgkinson, Stephen Staines, Cormac Browne, Stuart McRobbie, Alex Wright, Ralph P. Tatam

**Affiliations:** 1 Engineering Photonics, Cranfield University, Cranfield, Bedfordshire, MK43 0AL UK; 2 TRL, Crowthorne House, Nine Mile Ride, Wokingham, RG40 3GA UK; 3 Present Address: WSP, King James VI Business Centre, Friarton Road, Perth, PH2 8DY UK; 4 Present Address: National Highways, Safety, Engineering and Standards, Guildford, GU1 4LZ UK; 5 Present Address: XAIS-PTS, Britannia House, 1 Rough Hey Road, Grimsargh, Preston, PR2 5AR UK

**Keywords:** Optical sensors, Infrared spectroscopy, Civil engineering

## Abstract

The development and experimental performance of instrumentation to measure ageing-related spectroscopic changes in bitumen is described. Oxidation of bitumen at the surface increases the number of carbonyl (C=O) bonds, and this can be measured in the 6 μm region (1700 cm^−1^) of the mid-infrared. Standoff measurements of surface reflectivity were performed using 4 discrete wavelengths, 3 for the carbonyl absorption and the fourth as a spectral reference. The standoff height of 20 cm caused problems resulting from the presence of numerous strong absorption lines of atmospheric water in the optical path, which was solved by use of wavelengths centred within available “water windows” and a pathlength-matched reference channel. The instrument was tested using bitumen samples aged artificially using UV exposure. Results illustrating the instrument’s response to bitumen age, along with tolerance to changes in height and tilt, are shown. Measurements made during preliminary field trials on outdoor asphalt are also demonstrated. Part 1 of this paper describes the scientific challenges involved in designing this instrument.

## Introduction

**Fig. 1:**
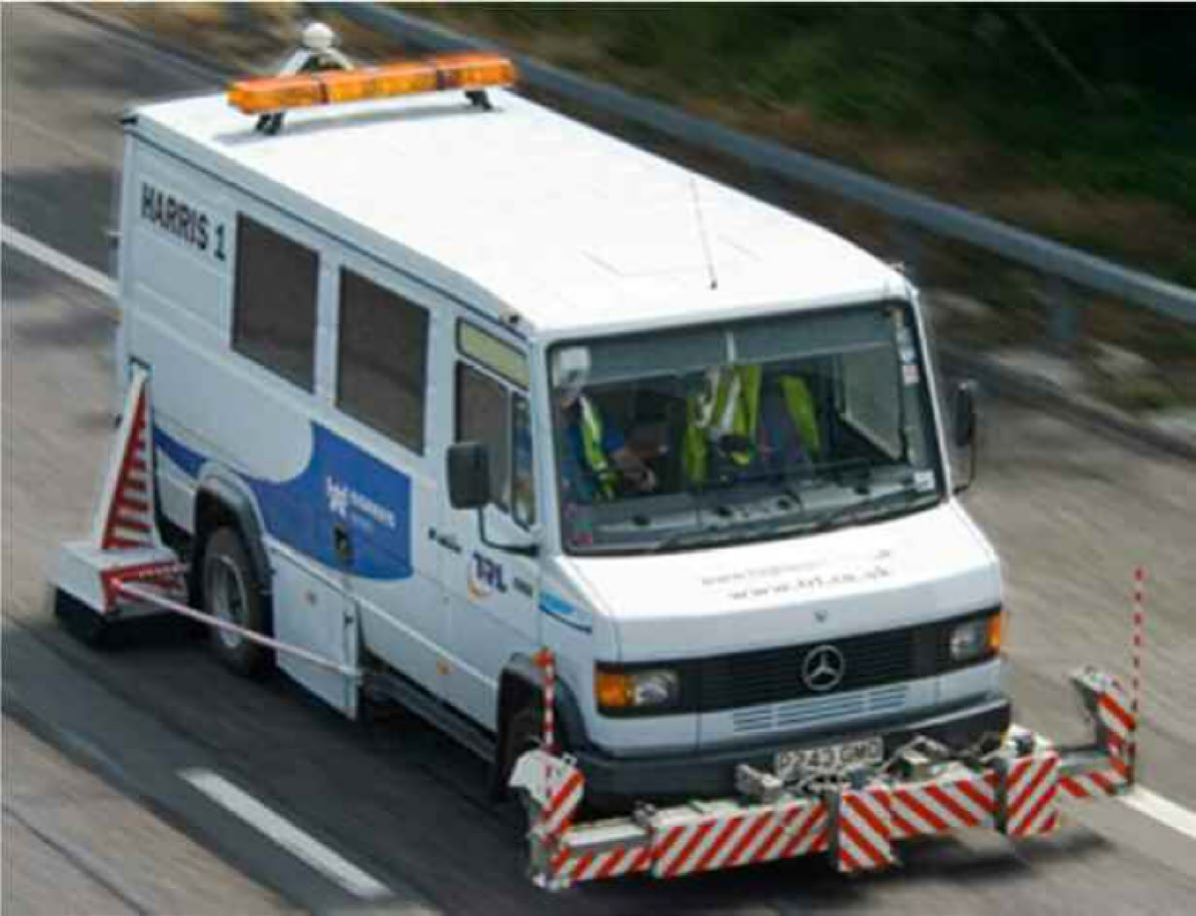
Traffic speed survey vehicle HARRIS1, showing laser bar on the front used to make measurements of surface texture via the deflection of projected laser beams. Taken from ^4^.

Management of high-value road networks such as England’s Strategic Road Network, consisting of motorways and strategic trunk roads, includes a rolling programme of measurement of pavement condition on both live and closed roads. Surveys monitor pavement shape, visual condition, structure and deflection under load, at high speed. The Traffic-speed Road Assessment Condition Survey (TRACS) approach was developed by the Highways Agency (now National Highways) in England and similar approaches are used for high-value road networks worldwide^1^. Traffic speed survey vehicles such as the Highways Agency Road Research Information System (HARRIS)^2^ are designed to operate at speeds of 50 mph/80 kph (~ 20 m/s) safely, without disrupting normal traffic significantly, so avoiding the need to close roads. Figure 1 shows an example of such a vehicle in use.

A key failure mode known as fretting (or ravelling) can occur when hardening, with embrittlement of the bitumen binder causing loss of aggregate from the asphalt^3^. If untreated, this can lead to rapid loss of the surface layer and large potholes. Existing methods to detect fretting rely on the appearance of measurable surface texture after the onset of surface deterioration. Texture is measured at traffic speed using projected laser lines whose deflection is monitored using high-speed cameras (16 kHz)^4^.

Asphalt applied as thin surfacing systems on UK strategic roads typically consists of a bituminous binder plus mineral aggregate of approximately 10–14 mm in size. Chemical changes in asphalt with ageing may precede the onset of measurable physical changes^5^, however, instrumentation is not currently able to measure these chemical changes at traffic speed. Core samples may be extracted from in-service roads while closed, then analysed in a laboratory. However, the pavement must be reinstated afterwards, and this method only permits a limited number of sampling points to be accessed. Closing roads is costly, adds to congestion and puts maintenance personnel at risk.

Ultra violet (UV)-induced oxidation is considered to be the primary chemical degradation mechanism of bitumen in asphalt and this is known to increase the number of carbonyl (C=O) bonds within the material^6^. The formation of carbonyl bonds (C=O) by oxidation of the hydrocarbons in bitumen has been proposed as a universal marker of ageing for different bitumen types, including virgin bitumen and bitumen modified by addition of crumb rubber (CR), warm mix additives (WMA) and sulfur^7^.

In the UK, National Highways has supported research into monitoring the progress of oxidation using mid-IR spectroscopy. Prior research has used a hand-portable Fourier Transform Infrared Spectrometer to measure changes in diffusely reflected spectral features associated with carbonyl and other bonds in artificially aged bitumen and on closed roads^8^. This work demonstrated that changes in the spectrum at the wavelengths associated with carbonyl features can be associated with UV-induced ageing of the bitumen. There is interest in making such measurements at traffic speed (20 m/s), which would enable practical monitoring of chemical ageing of road surfaces.

There are numerous applications in which rapid measurement of material properties is required in a non-contact, standoff geometry. Optical spectroscopy is well suited to this mode of operation for applications including recognition of polymers^9^, product quality control^10^ and detection of hazardous materials including explosives^11^.

Near infrared (NIR) spectroscopy is established as a powerful tool for rapid standoff monitoring of materials and products including food^12,13^, plants^14^ and crops^15,16^, soils^17^, paintings^18,19^ and more. The availability and acceptable cost of charge-coupled device (CCD) spectrometers and hyperspectral imaging has enabled widespread use of this technique in this region of the spectrum, despite relatively low strength spectral features.

In the mid-infrared (mid-IR) region, spectral absorption is higher and new technology, especially the use of tunable lasers, is enabling the measurement of spectra in standoff mode. Standoff measurement of the diffuse reflectance of pharmaceuticals has been reported at a distance of 15 cm using an external cavity quantum cased laser (EC-QCL), with 4 cm^−1^ resolution over the range 1600–990 cm^−1^^20^. Incident and collected beams were normal to the surface and humidity was controlled, but present (34% RH at 20 °C). IR imaging of trace substances has been achieved in the 6–11 μm region using an external cavity quantum cased laser (EC-QCL) and a focal plane array detector, with full spectral sweeps across this region performed within 25 s^21^. Diffuse reflectance spectra have also been obtained using a dual comb spectrometer (DCS) source, to identify different trace materials within a timescale of 20 ms^22^. The DCS was able to scan approximately a 100 cm^−1^ region of the spectrum centred around 1210 cm^−1^ (8264 nm), with a linewidth of 0.0003 cm^−1^ and line a separation of 0.3 cm^−1^.

The two papers in this series describe work to develop a novel instrument to make standoff measurements of asphalt reflectivity in the spectral region corresponding to carbonyl absorption, in a format compatible with traffic speed operation. The aim of this instrument is to be able to quantify the level of oxidation of bitumen, and thereby develop techniques to predict the onset of deterioration of road pavements. In part 1, the spectroscopic basis for this measurement and instrument design decisions driven by operational requirements^23^ is considered. In part 2, the development of the instrumentation is described, along with results of testing with bitumen subject to accelerated ageing via intensified oxidation through UV exposure. The instrument was mounted on a trailer for use at walking speed and used to make preliminary measurements on outdoor asphalt. The ultimate aim is for the instrument to be transferred to a traffic speed trailer, to complete preliminary measurements on roads at traffic speed.

Although measurements of carbonyl absorption are well-established in laboratory analysis as a measure of carbonyl ageing, to date there is little work to link this to measurements of surfaces *in-situ*, with studies that have been undertaken performing measurements from bitumen extracted from core samples^24^. A study using a hand-held Fourier transform infrared (FTIR) spectrometer was able to establish a correlation between bitumen ageing and spectral absorption in the 1700 cm^−1^ region, and to identify associated absorption features on in-situ road surfaces^7^. However, the instrument was unable to make measurements that were swept across the entire surface and therefore could not be used to survey roads at scale. The instrument described here represents the next step, to allow users to test the hypothesis that standoff measurement of carbonyl absorption can be used to monitor the chemical ageing of roads.

## Operational requirements and critical test parameters

Part 1 of this series outlined the rationale behind critical performance requirements for this instrument^23^. To summarise, the following are required, and this formed the basis for the test programme:

- A standoff distance of 20 cm is needed for the instrument to cope with changes in pavement level on in-service roads. Height changes of the pavement with respect to the instrument can be reduced to ± 1 cm by use of a servo mechanism, whereby a separate displacement laser is used to measure the distance of the instrument from the surface with a servo system utilised to maintain the distance of the instrument from the surface within the required range. Combined with the axle width of the proposed installation on a trailer, this also imposes a need to cope with a tilt of ± 15°.
- To measure bitumen ageing, the instrument must be able to measure changes in reflectance of R ~ 0.1, and these must be stable over time. This requires a noise-equivalent reflectance change of R ~ 0.01.
- Measurements should be averaged over a distance of 10 m, corresponding to a period of 0.5 s if operated at traffic speed (20 m s^−1^).
- The instrument should not be perturbed by changes to the level of atmospheric water vapour in the optical path.
- Operation in dry surface conditions is required, and not onerous as many existing instruments also have this requirement.

An instrument was proposed based on measurement of asphalt reflectivity from 4 lasers, co-aligned to a single spot to be moved along the road surface so as to scan along a single line in the direction of travel^22^. Three of these lasers would make measurements at chosen wavelengths across the 6 μm region, and one would make a reference measurement at a wavelength substantially unaffected by ageing. Measurement would take place by modulating and demodulating the laser intensities at 4 different (separable) frequencies, so as to eliminate direct current (DC) voltage changes in intensity caused by, for example, background radiation.

Operation at traffic speed also imposed a need for high speed (quasi-simultaneous) spectral acquisition so that the natural granularity of the surface would not result in an unstable spectral baseline. This was achieved for discrete lasers by using modulation frequencies *f* of *f* ≥ 20 kHz, since this corresponds (at traffic speed) to 10 modulation cycles made across a 10 mm section of aggregate, this representing a characteristic scale of asphalt’s granularity. Frequencies of f ≥ 40 kHz therefore had to be avoided so that cross-talk in the demodulator at higher harmonics would not perturb the measurement.

Careful referencing was required for two reasons:

(i) The reference wavelength referred to above was used to compensate for overall changes to instrument throughput and bulk surface reflectivity that might be caused by dirt or degradation on optical surfaces, changes to the responsivity of the detector and changes to overall reflectivity caused by changes to the height or tilt of the surface^25^.
(ii) A reference path (for all 4 beams) was used to compensate for the non-zero residual optical absorption by water vapour that remained despite operation in so-called water windows. The level of water vapour in the optical path was potentially highly variable as a result of vehicle exhaust turbulence and temperature changes.

This results in a total of 8 detection channels as illustrated in Table 1.

**Table 1:**
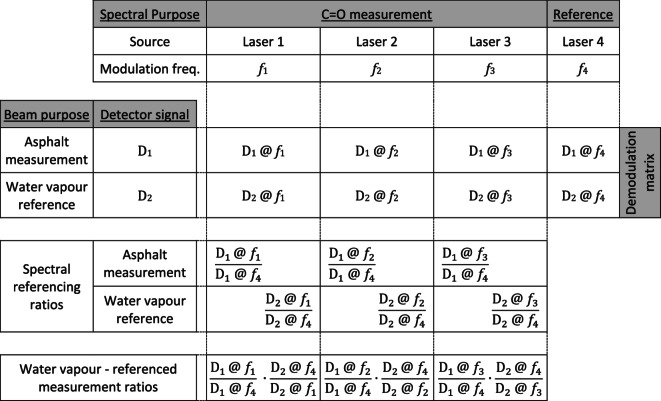
Modulation and demodulation scheme across 8 detection channels, followed by data analysis to provide spectral and water vapour referencing.

A more in-depth description of the referencing is presented in part 1 of this series ^23^.

## Laser choice and characterisation

Laser wavelengths were chosen to align with water windows and to provide measurements that would span the C=O region to provide indications of ageing as discussed in Part 1 (see Fig. 4, part 1). They were also constrained by commercial laser availability; there is limited choice in the 5–7 μm region because the ubiquitous presence of water vapour means that there is little demand for sources (or indeed other technology) operating in this region. Continuous-wave (CW) quantum cascade lasers (QCLs) were available with emission above around 4.3 μm and interband cascade lasers (ICLs) were available to cover the reference region. Table 2 shows the final laser wavelengths and their operating parameters.

**Table 2:** Chosen laser details: emission wavelengths and drive/modulation parameters.

Purpose	Reference	C=O Measurement		
Centre wavelength/nm	3797.0	5776.0	6092.4	6277.5
Centre wavenumber/cm^−1^	2633.6	1731.3	1641.4	1593.0
Type (manufacturer)	ICL (Nanoplus)	QCL (Alpes)	QCL (Alpes)	QCL (Alpes)
Collimation option	External to laser package	Internal to laser package	Internal to laser package	Internal to laser package
Operating temperature/°C	30	42	30	25
DC injection current/mA	55	383	302	231
Modulation current/mA	± 25	± 15.5	± 13	± 17
Modulation amplitude/cm^−1^	± 2.02	± 0.29	± 0.33	± 0.50
Modulation amplitude/nm	± 2.54	± 0.98	± 1.22	± 1.97
Modulated power/mW RMS	6	9	16	21
Modulation frequency/kHz	36	40	23	28

**Fig. 2:**
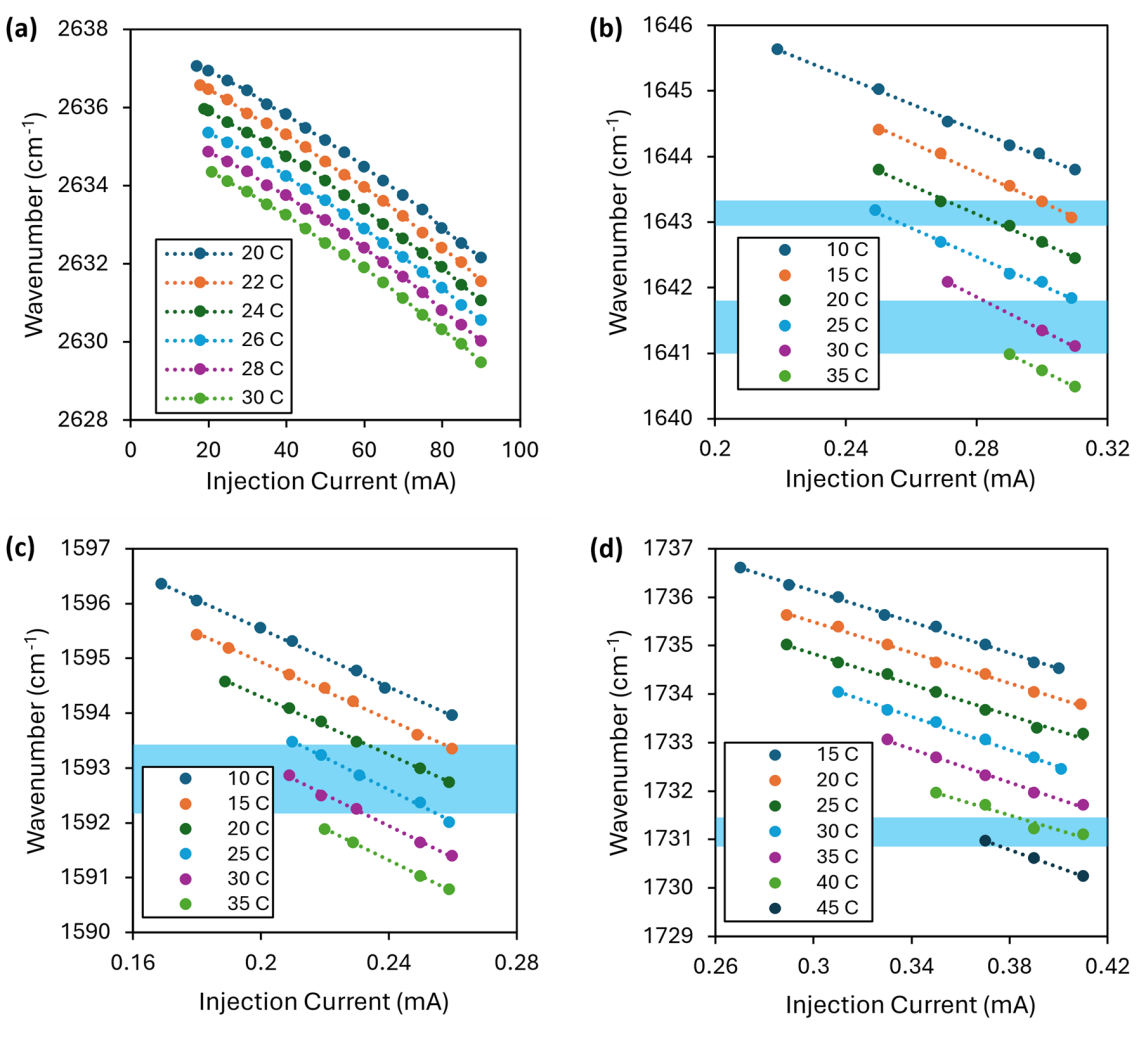
Tuning characteristics of (**a**) the reference ICL and (**b**–**d**) the 3 measurement QCLs at different laser temperature setpoints. The shaded bars on the QCL graphs indicate the locations of the water absorption windows. The tuning curves illustrate the degree to which the width of the water windows limited the accessible level of current modulation.

As described in Part 1 of this paper, to avoid interference from water vapour absorption, the lasers had to be modulated so that their emission wavelength remained within the windows between the absorption lines. Each laser was therefore fully characterized using a Thorlabs OSA207 mid-IR optical spectrum analyser (OSA) to provide the precise tuning ranges that could be utilised, as shown in Fig. 2. The bars across each graph indicate the absorption window within which the lasers were able to be modulated.

**Fig. 3:**
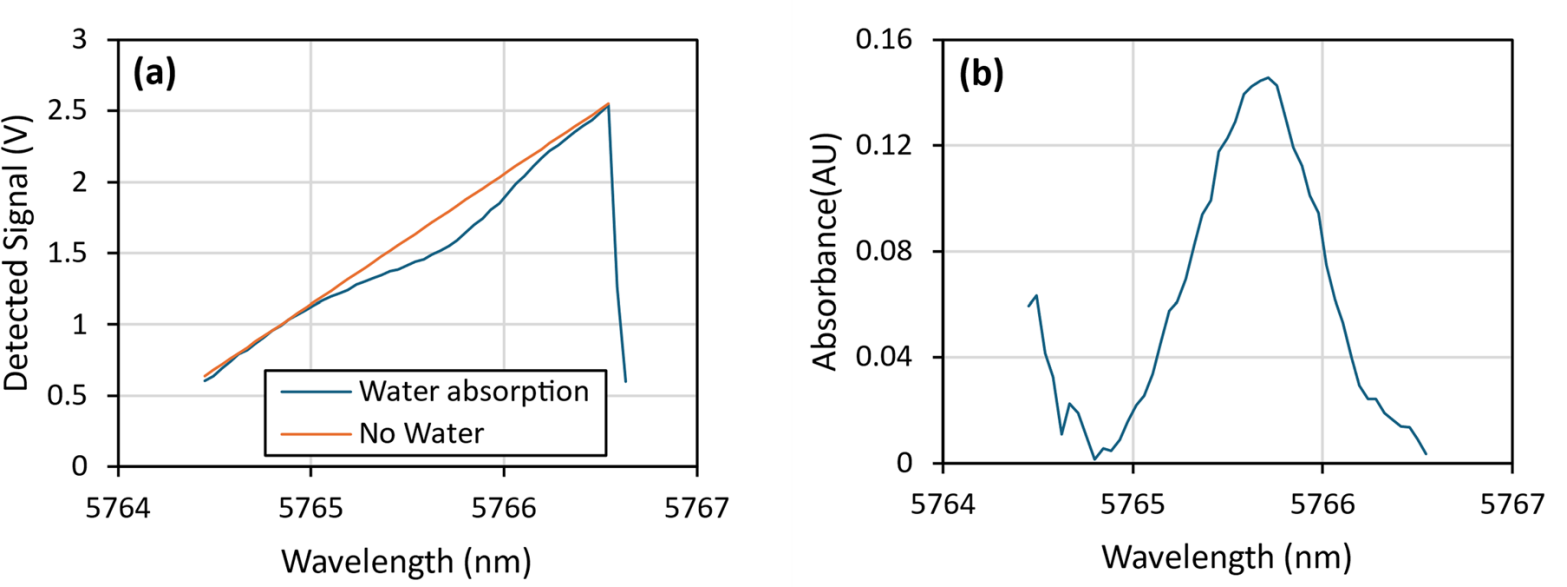
(**a**) Received signals measured during a spectral scan created via a current ramp, measured in laboratory air using a pathlength of approximately 5 cm, and a nominally 5776 nm (1731 cm^−1^) QCL. The calculated absorbance (**b**) shows the presence of a water absorption line, confirmed to be at 5765.6 nm (1734.4 cm^−1^), as expected using the current and temperature tuning parameters of Fig. 2.

A key risk was identified, namely that the spectral location of water lines would not be as expected. Since very high spectral resolution is required to operate within the identified water windows, a minor lack of accuracy, in either the measurements or the spectroscopic data used in planning, could cause a problem. Figure 3 shows the measurement of a strong water line (~ 75% RH at 21 °C) in laboratory air over a pathlength of approximately 5 cm. Its wavelength was measured using the laser tuning parameters required to access it and the plots of Fig. 2, therefore the wavelength values derive from a laboratory OSA. The centre wavelength of the water line was confirmed to be 5765.6 nm (1734.4 cm^−1^) using the OSA, which agrees well (to within the 0.8 nm/0.25 cm^−1^ resolution of the OSA) with the expected value. Thus, agreement between expected and laboratory wavelength scales was generally confirmed.

This measurement also confirmed the requirement to operate within the water absorption windows. With the measured signal subtracted from a theoretical baseline, as demonstrated in Fig. 3, a significant change in signal intensity caused by the presence of water vapour can be observed. If the system was operated over this wavelength range, any variations in the background atmospheric water vapour concentration would significantly affect the reliability of the measurements made.

## System architecture

**Fig. 4:**
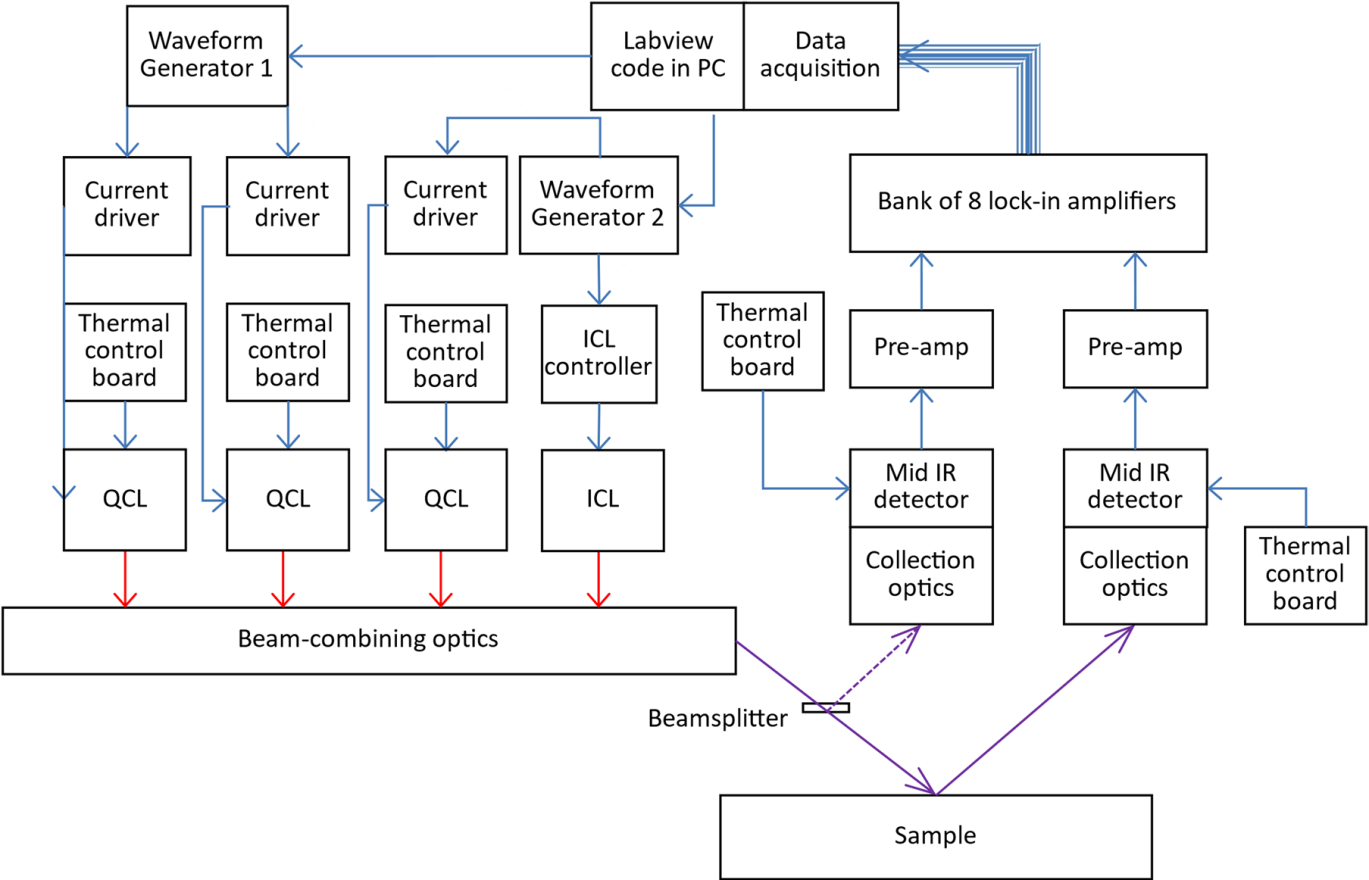
Schematic diagram of the instrument system architecture.

Figure 4 illustrates a schematic line diagram of the system architecture. Two independent waveform generators (Digilent Analog Discovery 2), controlled via a LabVIEW-based data acquisition and control system, were used to send four different sinusoidal modulation frequencies (23 kHz, 28 kHz, 37 kHz, 40 kHz) to the four laser controllers (3 × Wavelength Electronics LD2.5CHA for the QCLs and 1 Thorlabs ITC102 for the ICL). The modulated laser outputs were then combined into a pseudo-collimated beam using an off-axis parabolic mirror and directed towards the target, with a portion of the beam split off using a wedged CaF_2_ window to provide a reference arm. The incident light was diffusely reflected from the target surface, and was then collected using a large (50 mm diameter) off-axis parabolic mirror and directed onto the detector (Hamamatsu P6606-320). For laboratory measurements the collected signal was then demodulated using a series of 8 lock-in amplifiers (a combination of 3 × SRS SR850, SRS SR830, SRS SR844, SRS SR865, EG&G 5205, and Femto LIA-BVD-150-H) before being recorded using a 16-bit data acquisition system (NI USB-6366 ADC). The significant settings for each lock-in amplifier and their corresponding laser and signal arm are displayed in Table 3.

**Table 3:** Significant settings for each lock-in amplifier used, alongside their respective laser and signal arm.

Laser	Signal arm	Lock-in amplifier	Lock-in amplifier settings		
			Sensitivity/mV	Expand/unitless	Time constant/ms
3797.0 nm (2633.6 cm^−1^)	Sensing	SRS SR865	200	10	10
	Reference	SRS SR850	100	1	10
5776.0 nm (1731.3 cm^−1^)	Sensing	SRS SR844	300	10	10
	Reference	Femto LIA-BVD-150-H	10	1	10
6092.4 nm (1641.4 cm^−1^)	Sensing	SRS SR850	200	10	10
	Reference	EG&G 5205	20	1	10
6277.5 nm (1593.0 cm^−1^)	Sensing	SRS SR850	100	10	10
	Reference	SRS SR830	20	1	10

**Fig. 5:**
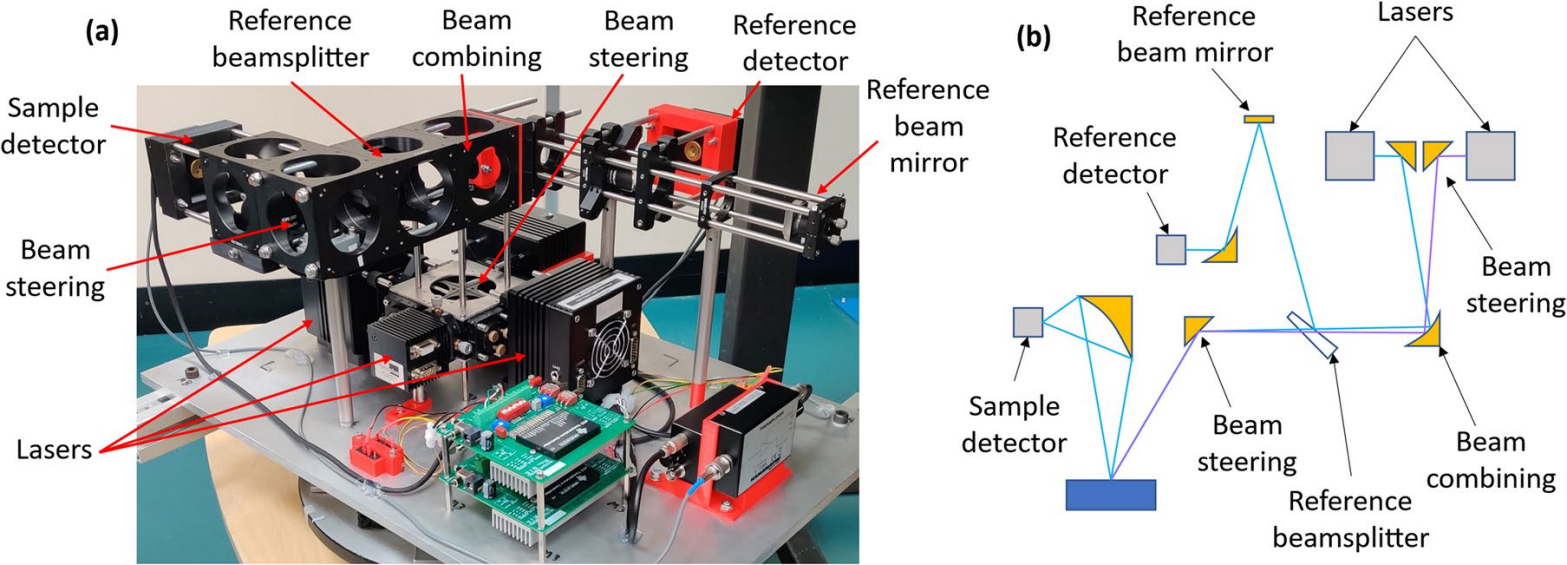
(**a**) Photograph of the instrument (dimensions 600 × 400 × 240 mm) shown upside-down to demonstrate components, alongside (**b**) a 2D representation of the optical setup (2 lasers not shown are arranged above and below the plane of the drawing).

To provide the required referencing, recorded signals from the QCLs were each divided by the signal from the ICL, before being referenced against the signals from the reference arm. The data processing scheme is illustrated in Table 1. Figure 5 shows the system implemented in a laboratory setting, alongside a simplified 2D diagram demonstrating the beam combination and collection optics. The instrument is suspended from an aluminium framework to allow measurement samples to be positioned underneath, similarly to a real-world scenario, such as measurement of road surfaces.

## Laboratory results

**Fig. 6:**
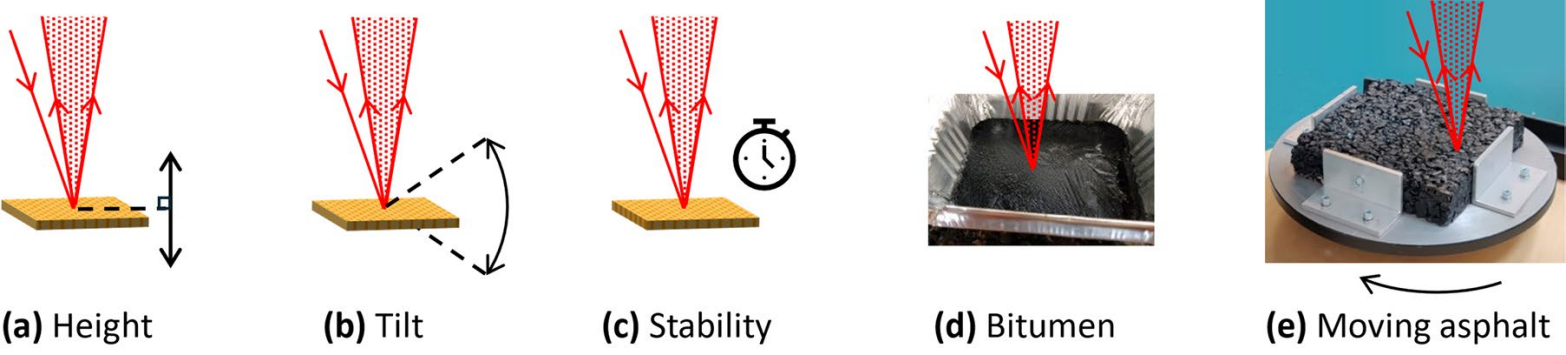
Simplified illustration of instrument tests, shown here for a diffuse reflection geometry. Tests (**a**–**c**) were performed using a calibrated Infragold sample, whereas (**d**) and (**e**) were performed using bitumen and asphalt samples prepared as described in the text.

Initial laboratory results were taken whilst the system was suspended on the aluminium framework. Measurements of the system’s response to changes in height and tilt, measurement stability, bitumen age and moving asphalt were undertaken. A diagram illustrating the individual measurements can be seen in Fig. 6. Each of these measurements were referenced against a measurement of an Infragold sample, taken at 0 mm height offset and 0° tilt. Infragold was utilised as a reference as it has a high (> 94%) and mostly uniform reflectivity across the mid-infrared^26^. This was done for all measurements in this paper to enable cross-referencing between measurements.

Three different orientations were tested to determine the optimum measurement geometry: specular reflection at 15°, diffuse reflection at 30°, and finally diffuse reflection at 15°. Initially, to test the suitability of each geometry, measurements of the system’s response to changes in height and tilt were undertaken.

### Specular reflection

**Fig. 7:**
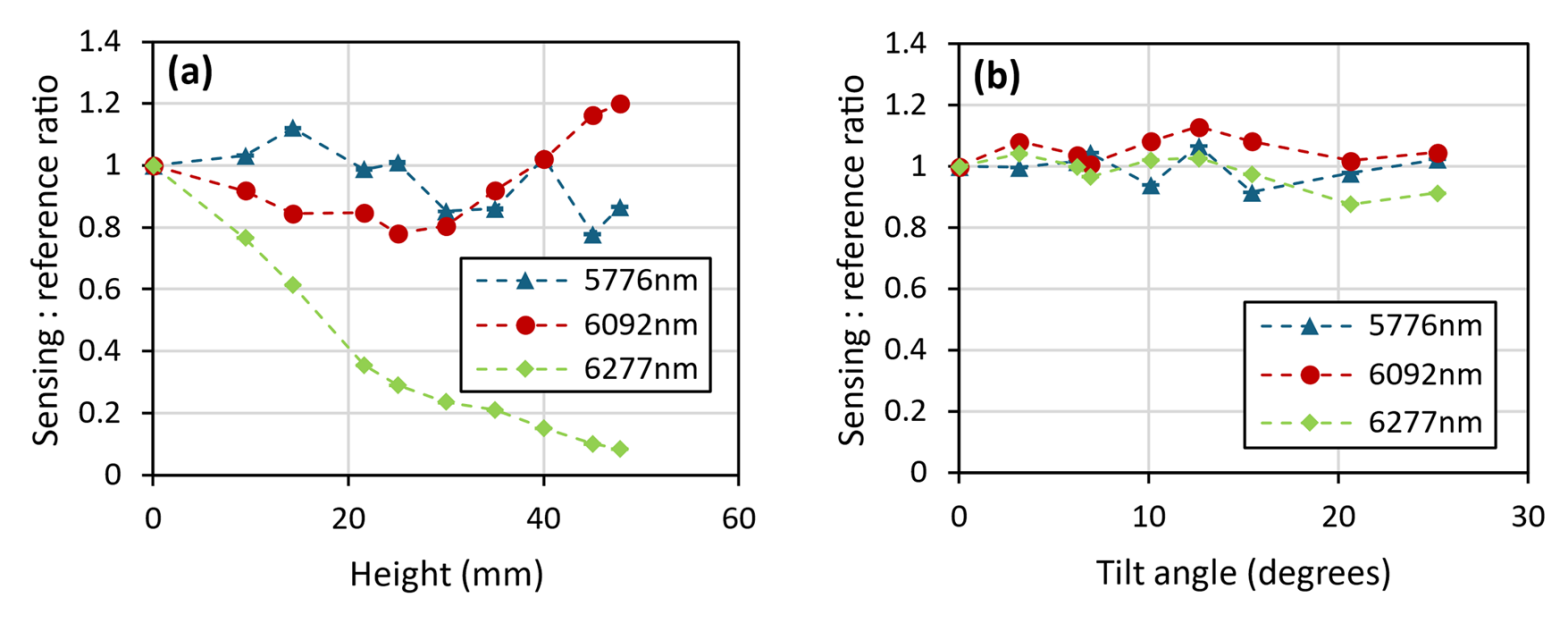
(**a**) Instrument response to changes in the height of the measurement point, and (**b**) response to changes in the angle of the sample at the measurement point, both for the 15° specular geometry. Dashed lines between points are used as a guide to the eye.

The first geometry, specular at 15°, was orientated so that the specularly reflected light was collected and averaged over 2 s (2000 measurement points) at 15° from the normal to the sample surface. To measure changes with height, a diffusely reflecting Infragold standard was positioned in the beam path on a height-adjustable stage. For tilt measurements, the Infragold standard was positioned at the zero-height point and its angle relative to the incident beam gradually adjusted. The collected results for these measurements are shown in Fig. 7, with the errors bars indicating the standard error in the mean across the measurement. The system’s response to changes in tilt at this geometry indicated that the instrument was tolerant to variation in surface angle up to at least 25°, however the response to changes in surface height show that there was a serious parallax issue with at least one of the lasers.

### 30° diffuse reflections

**Fig. 8:**
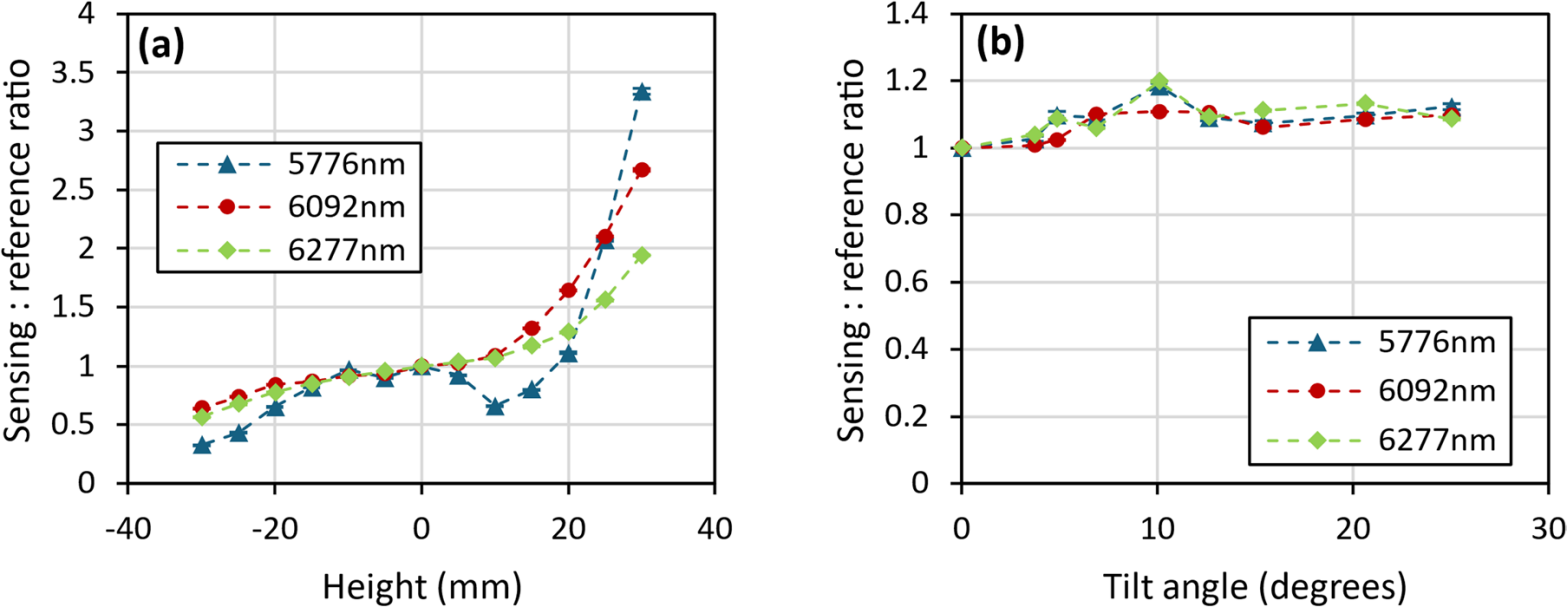
(**a**) Instrument response to changes in the height of the measurement point (with increased y-axis scale to demonstrate parallax issue) and (**b**) response to changes in the angle of the sample at the measurement point, for 30° diffuse geometry.

The instrument was re-orientated into a configuration that would allow measurements of diffusely collected light to be undertaken. Initially, a 30° angle of incidence was utilised, with the diffusely scattered light collected directly above the sample location, along an axis normal to the surface. The measured response to height and tilt changes, as shown in Fig. 8, demonstrate that there was a notable parallax issue with the height tolerance, as well as a small change in response for changes in the sample angle.

### 15° diffuse reflections

**Fig. 9:**
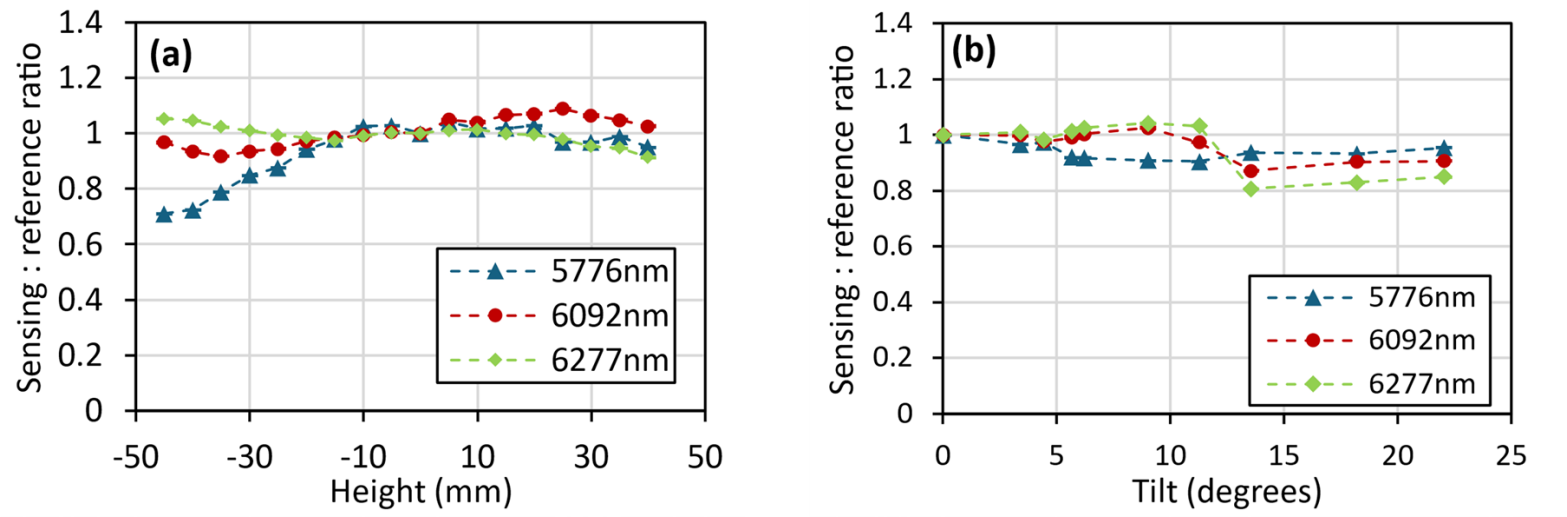
(**a**) Instrument response to changes in the height of the measurement point and (**b**) response to changes in the angle of the sample at the measurement point for 15° diffuse geometry.

The instrument was finally re-orientated into a configuration to allow measurements of diffusely reflected light with a 15° angle of incidence and collection normal to the surface. The results for height and tilt, as shown in Fig. 9, indicate that the instrument was tolerant to changes in height of ± 10 mm and a variation in surface angle of at least 5°.

From the different geometries that were investigated, the 15° diffuse orientation provided the best response for height and tilt measurements. As a result, this orientation was utilised for all remaining investigations.

To determine how the system responded to bitumen as it ages, six samples of fresh bitumen were produced and subjected to accelerated aging for 1 to 6 weeks in a UV chamber. 40/60 penetration grade bitumen (provided to the project as an example of binder typically used in Stone Mastic Asphalt (SMA)) was used for the ageing and 150 g of this bitumen was poured into each of six tins of size 100 × 40 mm, where it was allowed to settle. The tins were placed in a chamber for accelerated UV ageing, containing eight T8 tube lamps, emitting at 368 nm and rated to 15 W. Each lamp had a measured intensity of 1350 μW/cm^2^ at a distance of 15 cm. The field of view for each sample was such that the total intensity experienced by the samples was between 5000 to 6750 μW/cm^2^.

**Fig. 10:**
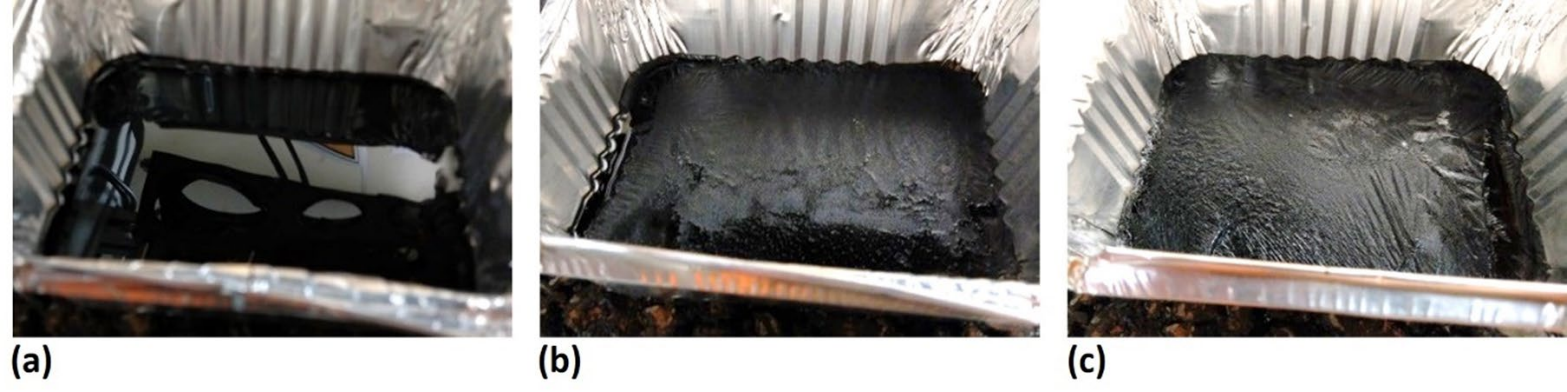
Examples of (**a**) fresh, (**b**) 2-week, and (**c**) 5-week UV-aged bitumen.

**Fig. 11:**
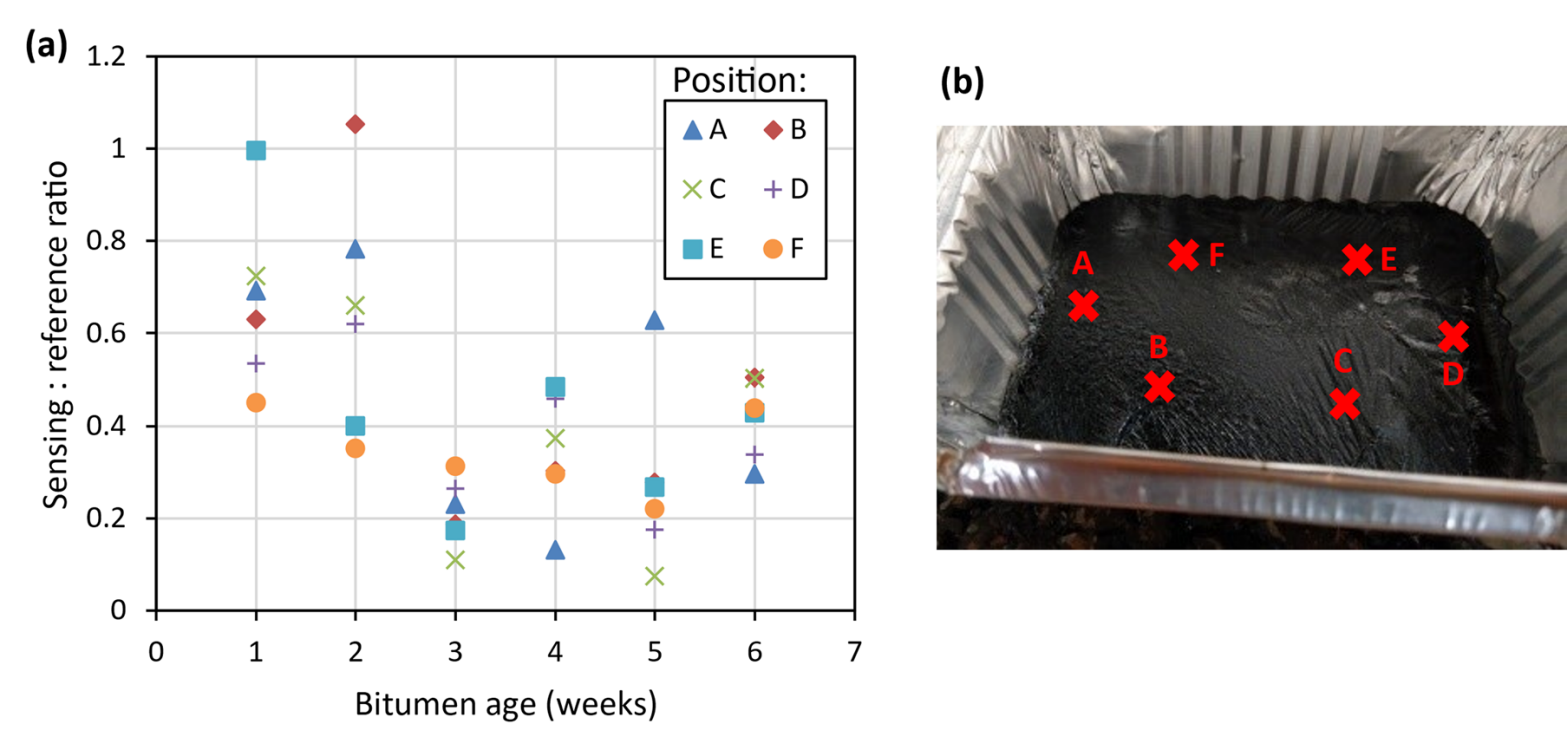
(**a**) Example of difference between signals recorded at different locations on each bitumen sample for the 6092 nm (1641 cm^−1^) QCL and (**b**) the sensing locations on one of the bitumen samples.

Examples of these samples can be seen in Fig. 10. Although prepared in Aluminium trays, it is not expected that the incident beams would be able to penetrate deep enough into the bitumen to reach the aluminium. Further detailed analysis of light penetration is described in part 1^23^. Two second measurements (2000 data points) were taken at six discrete locations on each sample, and at identical locations for repeated measurements made at different times. Measurement locations were identified by aligning to marks made on the bitumen tray, giving a location repeatability estimated to be ± 5 mm. The 6 measurements were averaged and referenced against a measurement taken of an Infragold sample. Figure 11 demonstrates the scatter observed across each bitumen sample for a single laser, alongside an image showing the approximate sensing locations on the sample surface. Due to the high specular reflection from the surface of the fresh bitumen samples (Fig. 10a), it was decided that measurements would not be made for this sample age. The nature of the surface meant that all incident beams would be reflected away from the collection lens, making any measurements of the surface unreliable.

**Fig. 12:**
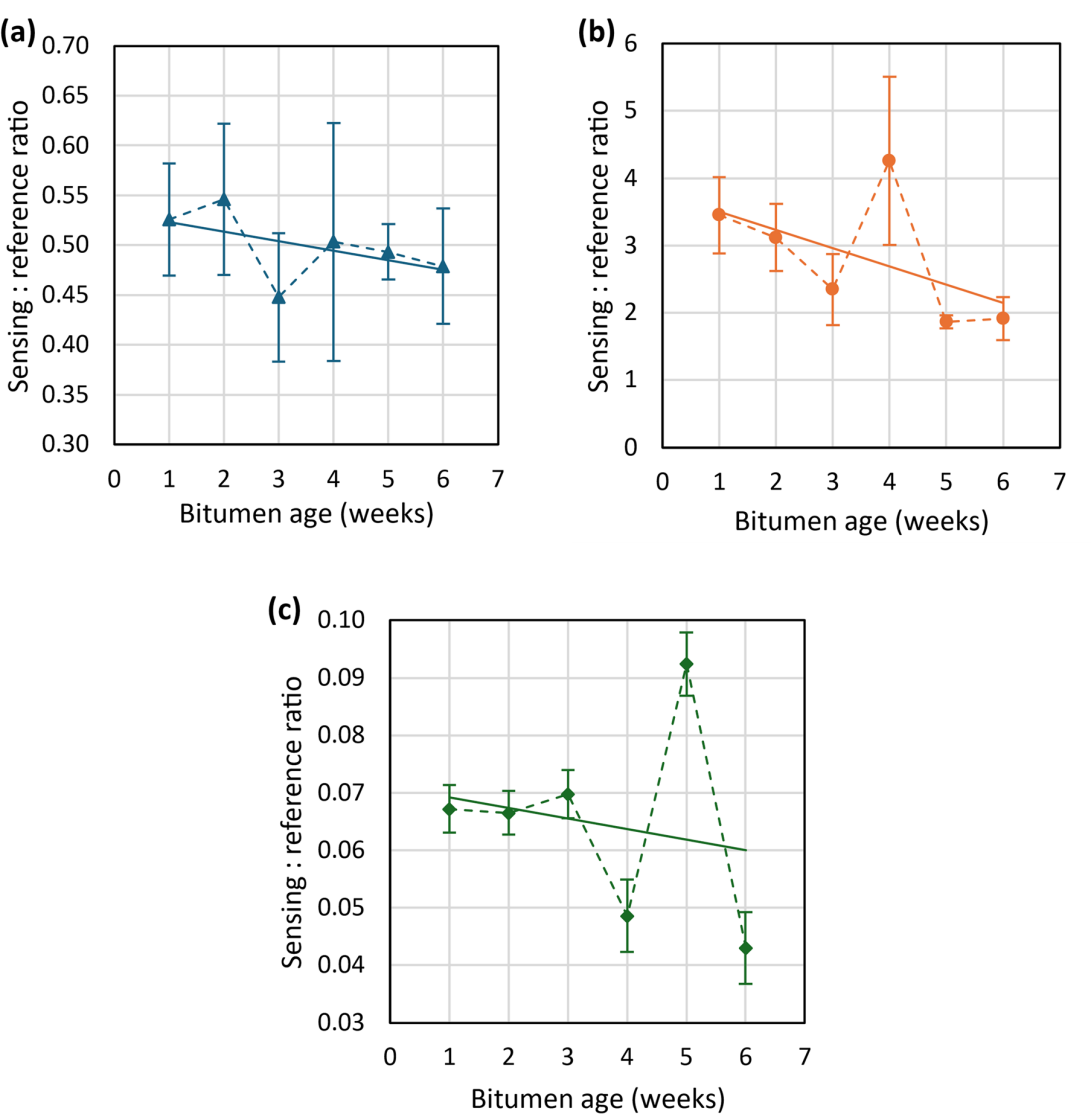
15° Diffuse reflection measurements of signal response of 1–6 week aged bitumen for (**a**) 5776 nm (1731 cm^−1^), (**b**) 6092 nm (1641 cm^−1^) and (**c**) 6277 nm (1593 cm^−1^).

The resultant graphs of aged bitumen measurements for each laser are shown in Fig. 12. The large error bars show the standard deviation for each measurement and are attributed to the inhomogeneity of the surface especially in the longer-aged samples. Increased levels of surface wrinkling with ageing can be seen in Fig. 10. The graphs show a trend in signal response for each laser with bitumen age, although the variability with measurement location is high. This downward trend was expected, due to the increase in the carbonyl absorption band as the bitumen ages reducing the reflected intensity (and subsequently collected signal strength) at these wavelengths.

**Fig. 13:**
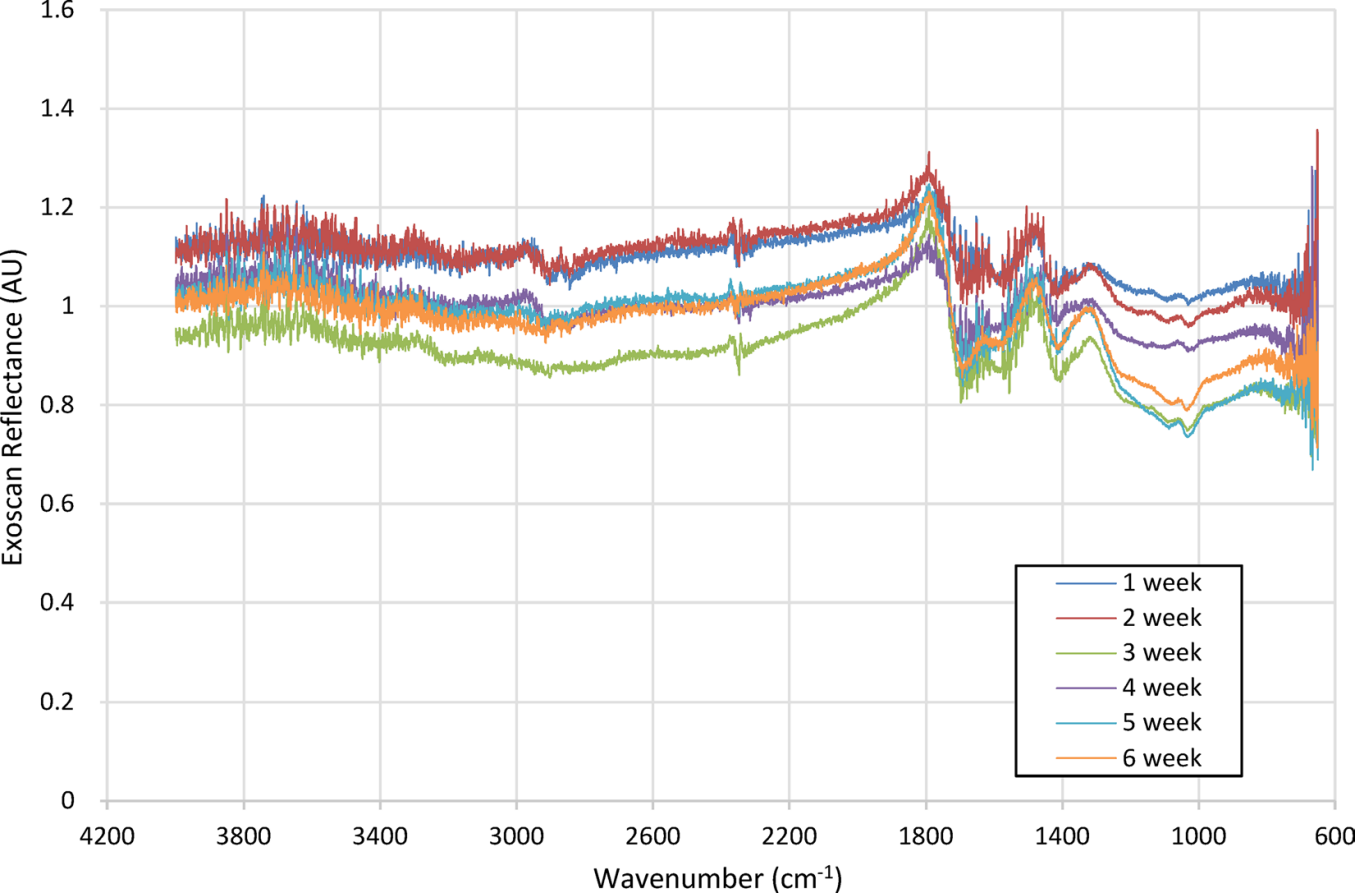
Reflectance spectra taken of each bitumen sample using the Exoscan 4100 handheld spectrometer.

To confirm that the instrument is providing a signal response that correlates with what is expected from a standard diffuse reflectance FTIR measurement, readings of each bitumen sample were taken using an Agilent 4100 Exoscan FTIR handheld spectrometer. Two measurements of each bitumen sample were performed, with each being referenced internally on the device against a measurement of a diffuse gold reflectance standard. Due to the size of the device, only two measurements could be reliably made per sample. The resultant averaged values of these measurements can be observed in Fig. 13.

**Fig. 14:**
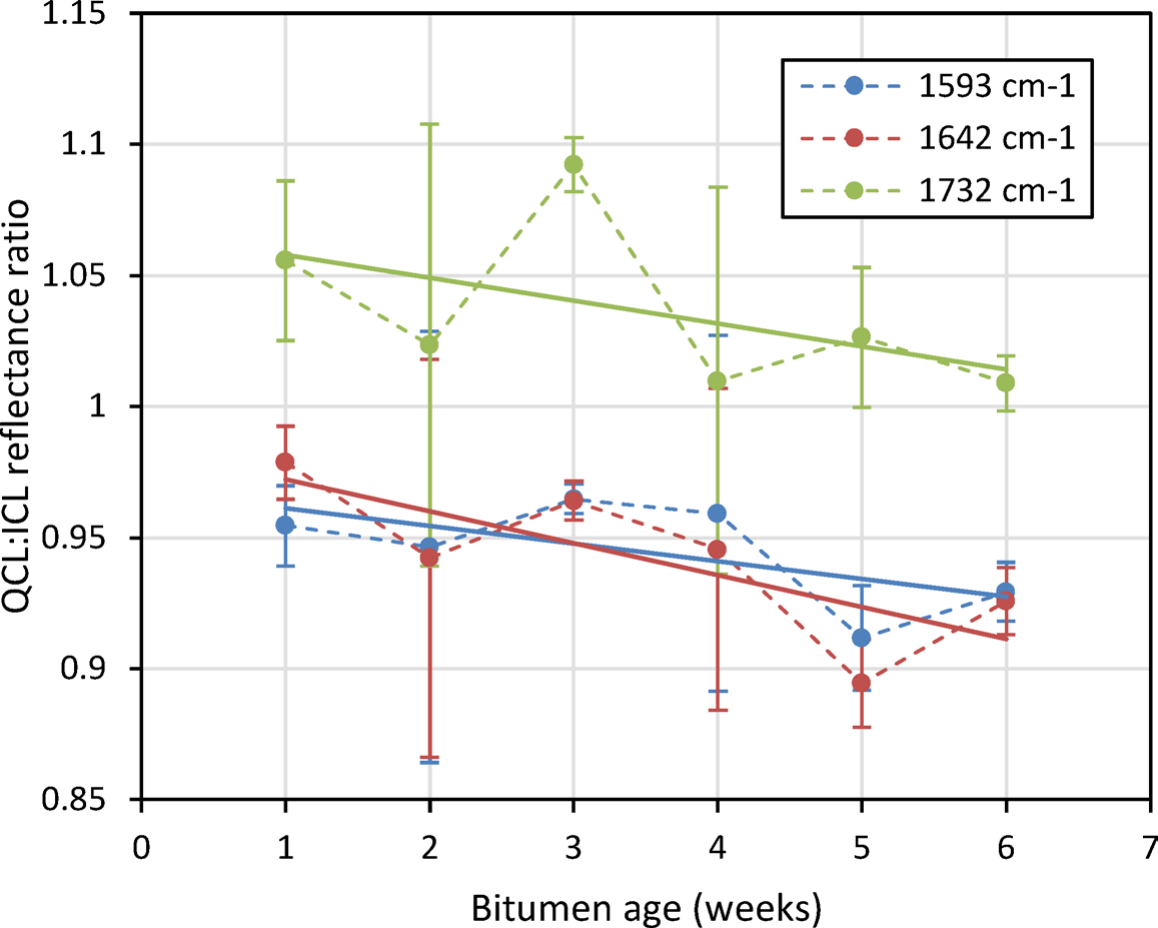
Referenced signal response for averaged wavelength ranges corresponding to instrument measurement laser wavelengths for each bitumen sample.

Taking the average intensity for the wavelength ranges corresponding to the measurement lasers and referencing them against the average intensity for the wavelength for the reference laser, response curves that match those made using the developed instrument could be obtained, as seen in Fig. 14.

The resultant curves demonstrate a signal response similar to that observed with the developed instrument. The error bars for these measurements are also on a similar scale to those seen in the measurements from the instrument, demonstrating that the heterogeneity and surface morphology of the samples is the cause of the scattering observed.

**Fig. 15:**
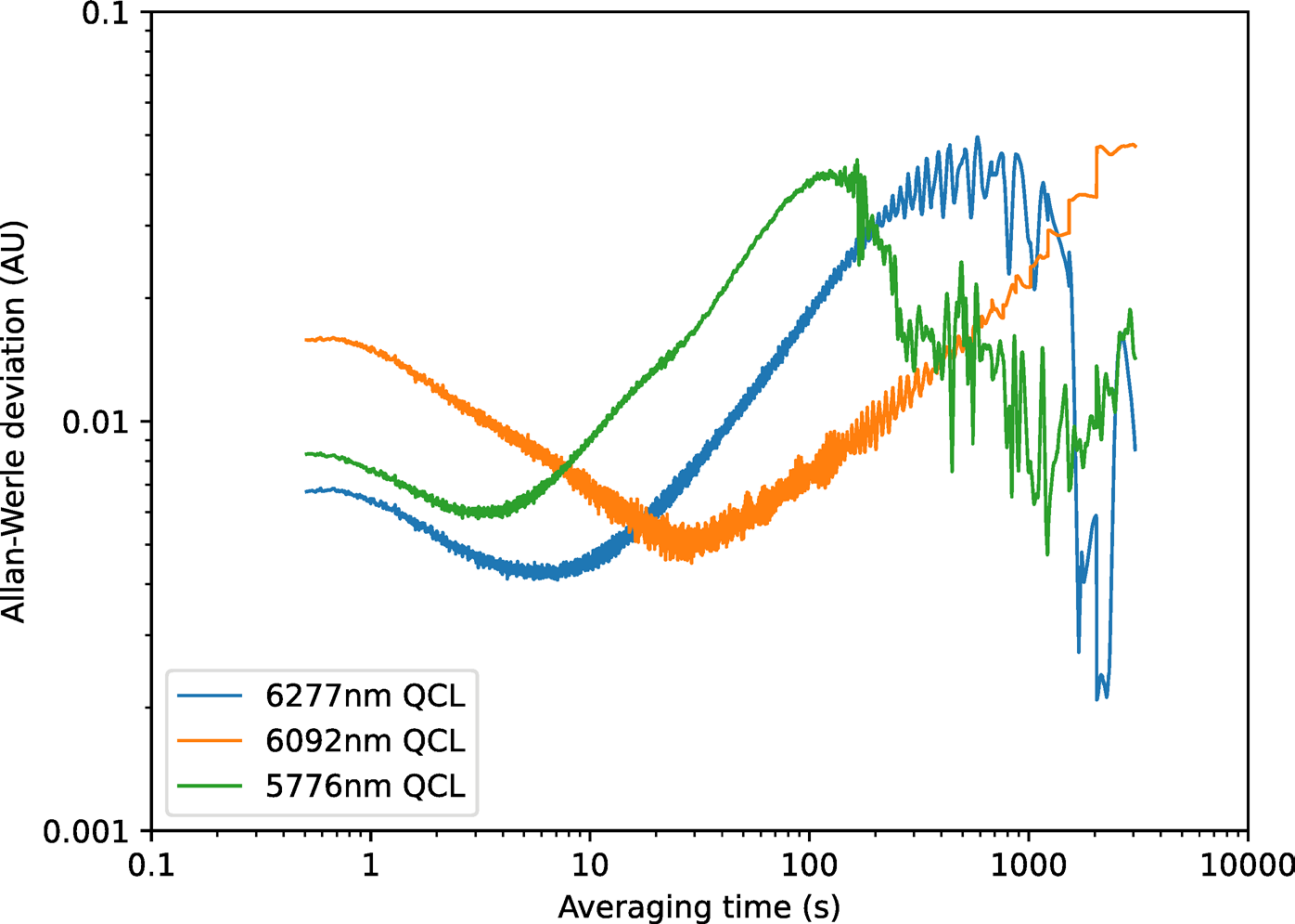
Allan-Werle deviation for diffuse reflection measurements of an Infragold standard using referenced QCLs.

**Fig. 16:**
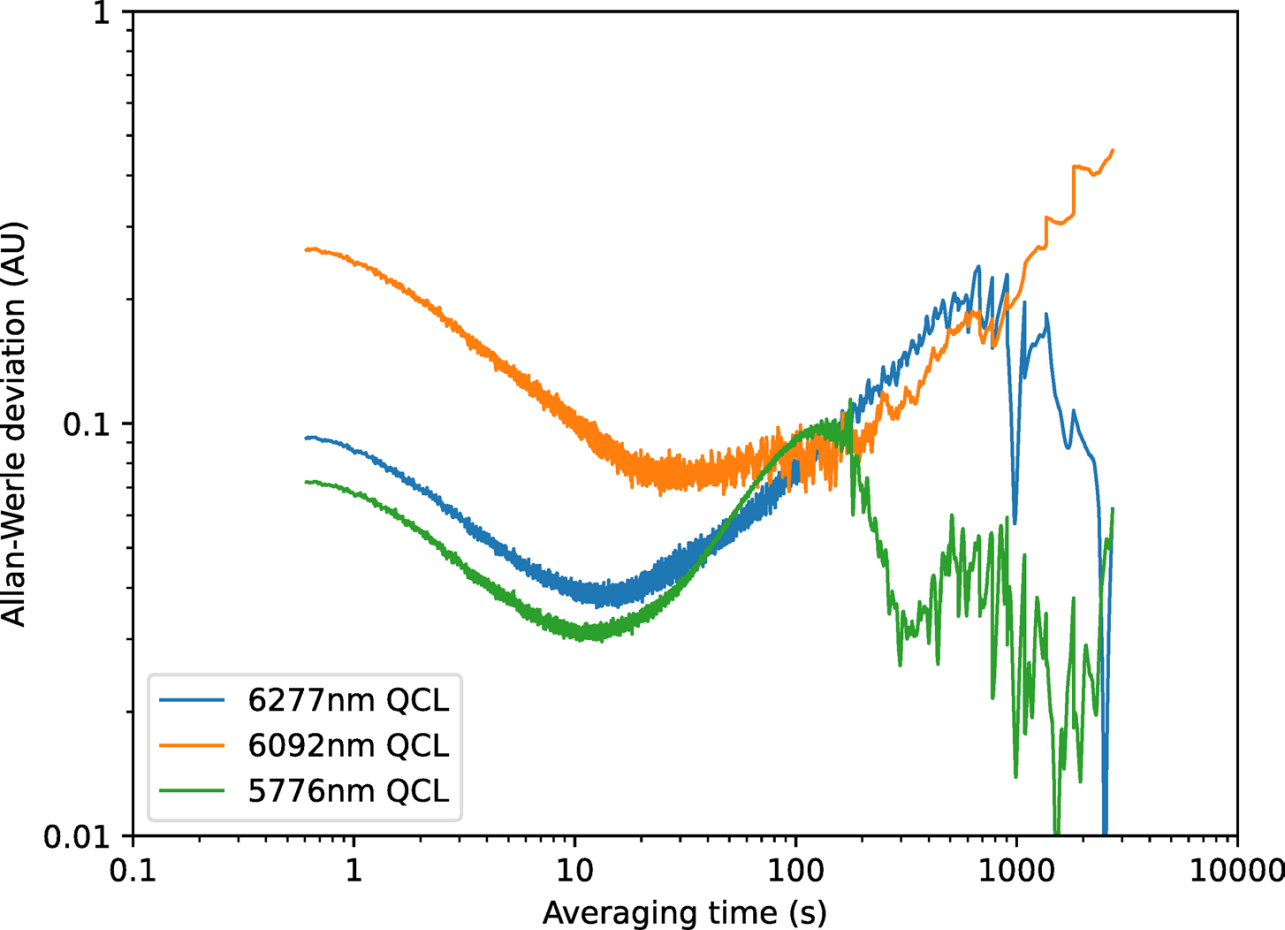
Allan-Werle deviation for diffuse reflection measurements of 1-week UV-aged bitumen using referenced QCLs.

To analyse long-term drift and stability, measurements were made of the diffuse reflection over a period of 40 min from two different samples, the Infragold sample and the 1-week UV-aged bitumen. The data were then analysed using the method of Werle et al. ^27^ to give measurements of the Allan-Werle deviation (the standard error in the measurement as a function of averaging time). The resultant curves can be seen in Figs. 15 and 16 respectively. These graphs indicate that minimum detectable levels of between 0.0045 and 0.006 can be measured on Infragold, with values of 0.03 to 0.08 possible when using bitumen, over averaging periods of 10 s. At an averaging period of 0.5 s the shape of the curves confirms that this region was dominated by white noise, most probably dominated by the detector, but a minimum detectable level of between 0.07 and 0.3 is still possible. The 6092 nm (1641 cm^−1^) laser does not appear to be as stable as the others, reaching a minimum at a longer averaging time and drifting for a longer period, however it is still possible to achieve a minimum detectable level for all three lasers below 0.01 for Infragold and 0.1 for bitumen.

**Fig. 17:**
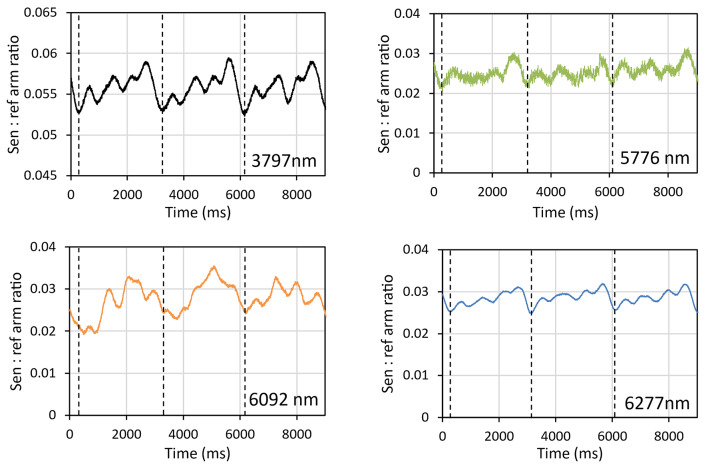
Measurements for each laser showing variation in signal observed on a rotating sample of asphalt at a linear speed of 0.2 m/s. Vertical dashed lines indicate the start of a new cycle.

As stated earlier, one of the main requirements of the instrument was for it to be able to operate at traffic speed. To determine how the instrument would respond to asphalt in motion, a 30 cm × 30 cm sample of asphalt was secured to a modified potter’s wheel and rotated at a constant rate of 0.35 Hz. The beam was directed at the outer edge of the rotating asphalt sample to simulate motion at approximately 0.2 m/s. Figure 17 shows the response from each laser for just over three full rotations (each rotation equating to approximately 50 cm of travel). The observed signal fluctuated partly as a result of being reflected differently as different angles of surface moved into and out of the beam, but also due to the beam moving between regions of bitumen and aggregate. No significant transient signals were observed that might result from the glinting of specular reflections and that could otherwise affect the response to diffuse reflections. Measurements from the 6092 nm (1641 cm^−1^) laser did not seem as repeatable as the other lasers, which is consistent with the previous findings on drift and stability.

## Mounting to trolley and results

**Fig. 18:**
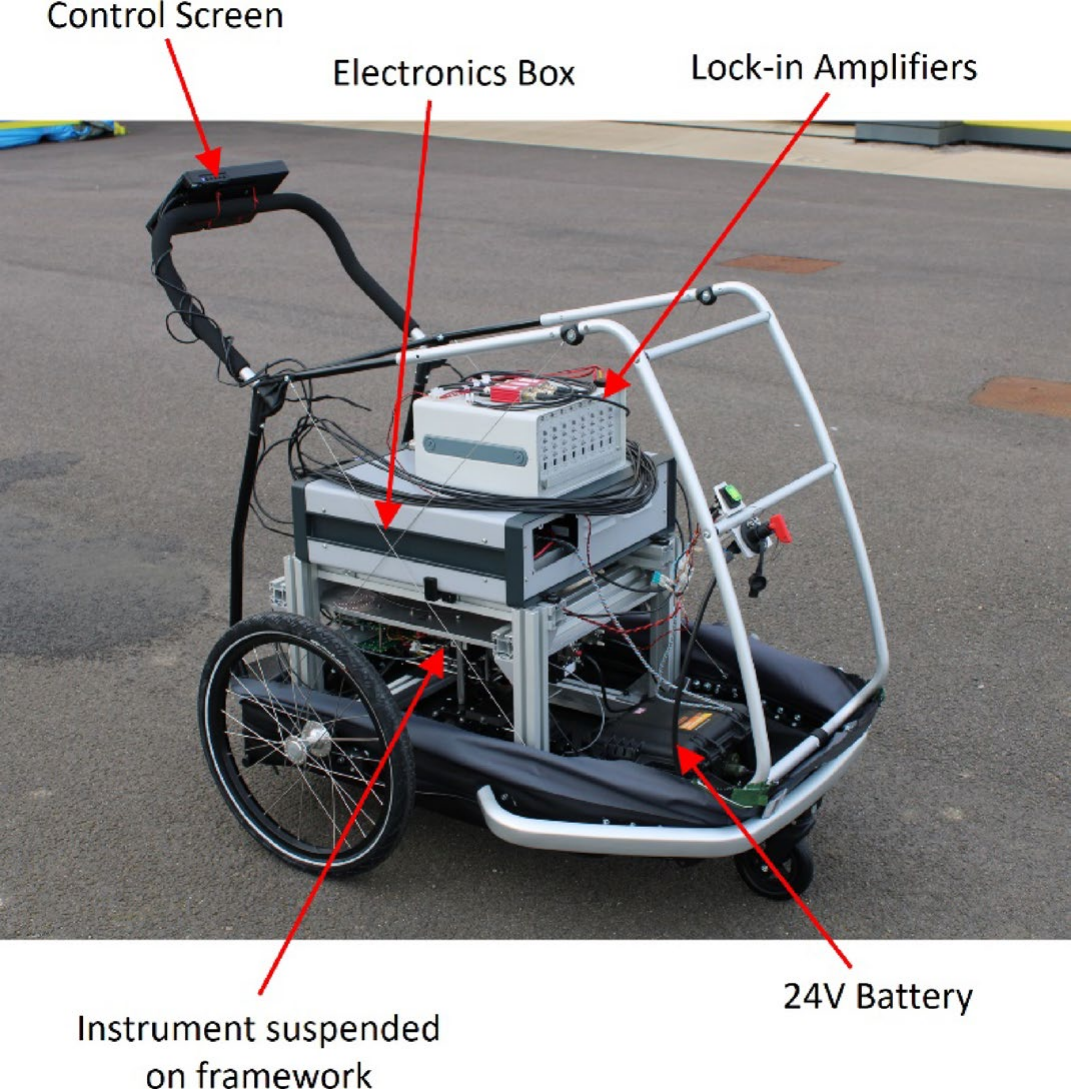
Fully assembled instrument mounted onto modified trolley (dimensions 115 × 86 × 103 cm).

The instrument was attached to an aluminium framework which in turn was mounted onto a hand-pushable trailer (a modified dog trailer, Croozer XXL) to allow outside measurements to be undertaken, as shown in Fig. 18. To accommodate the need for portability, the benchtop lock-in amplifiers were replaced by a bank of 8 lock-in amplifier boards (Femto LIA-BV-150-H), with the system powered by a large 24 V battery (Tracer 24 V 20Ah LiFePO4 Peli Case Battery Pack) and controlled via a touchscreen attached to the trailer handle. The battery was able to power the instrument for approximately 4 h.

To confirm the instrument’s performance after mounting, a number of the measurements previously undertaken, namely height, tilt, aged bitumen response and Allan-Werle deviation on aged bitumen, were repeated.

**Fig. 19:**
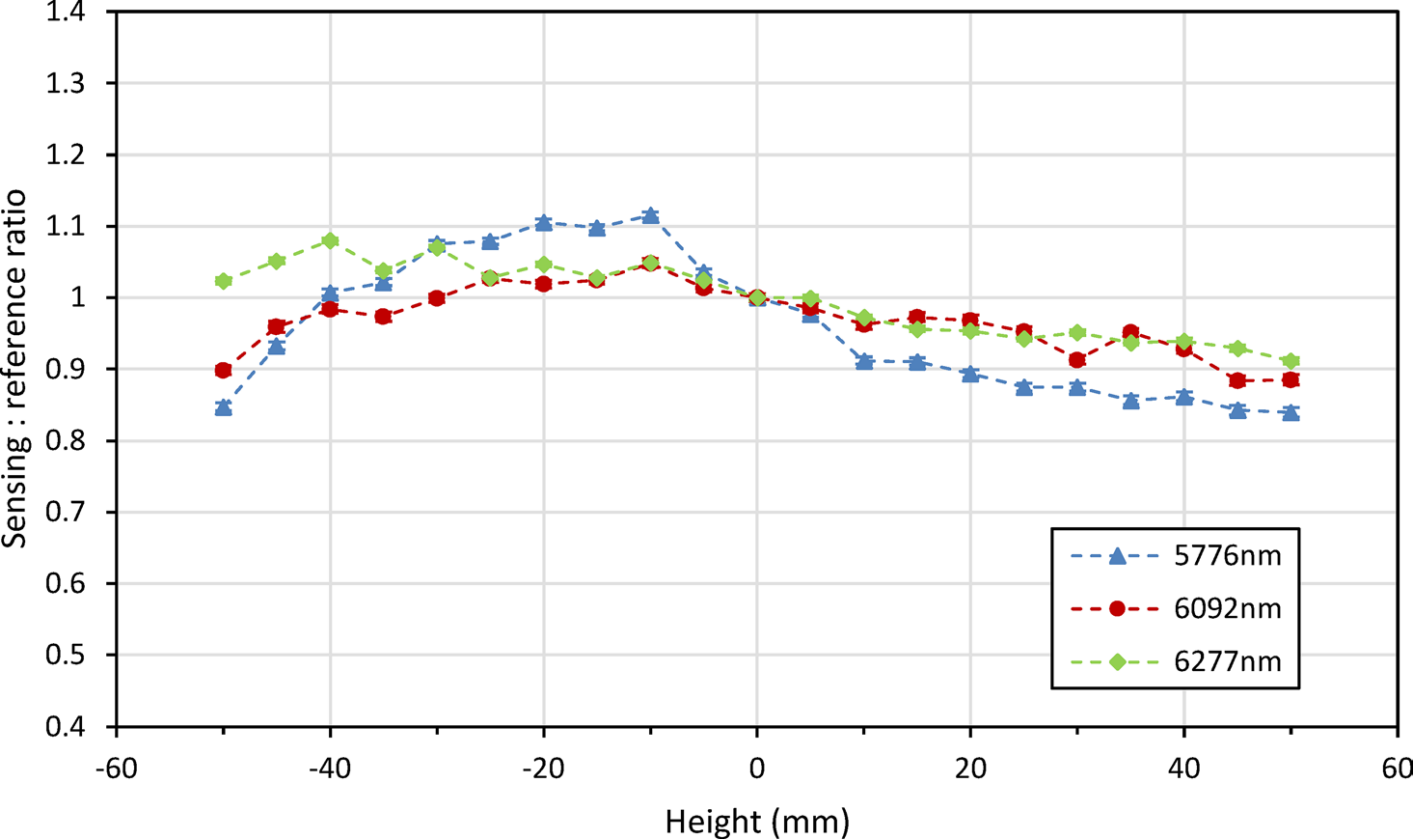
Instruments response to changes in the height of the measurement point after being mounted onto the modified trolley.

**Fig. 20:**
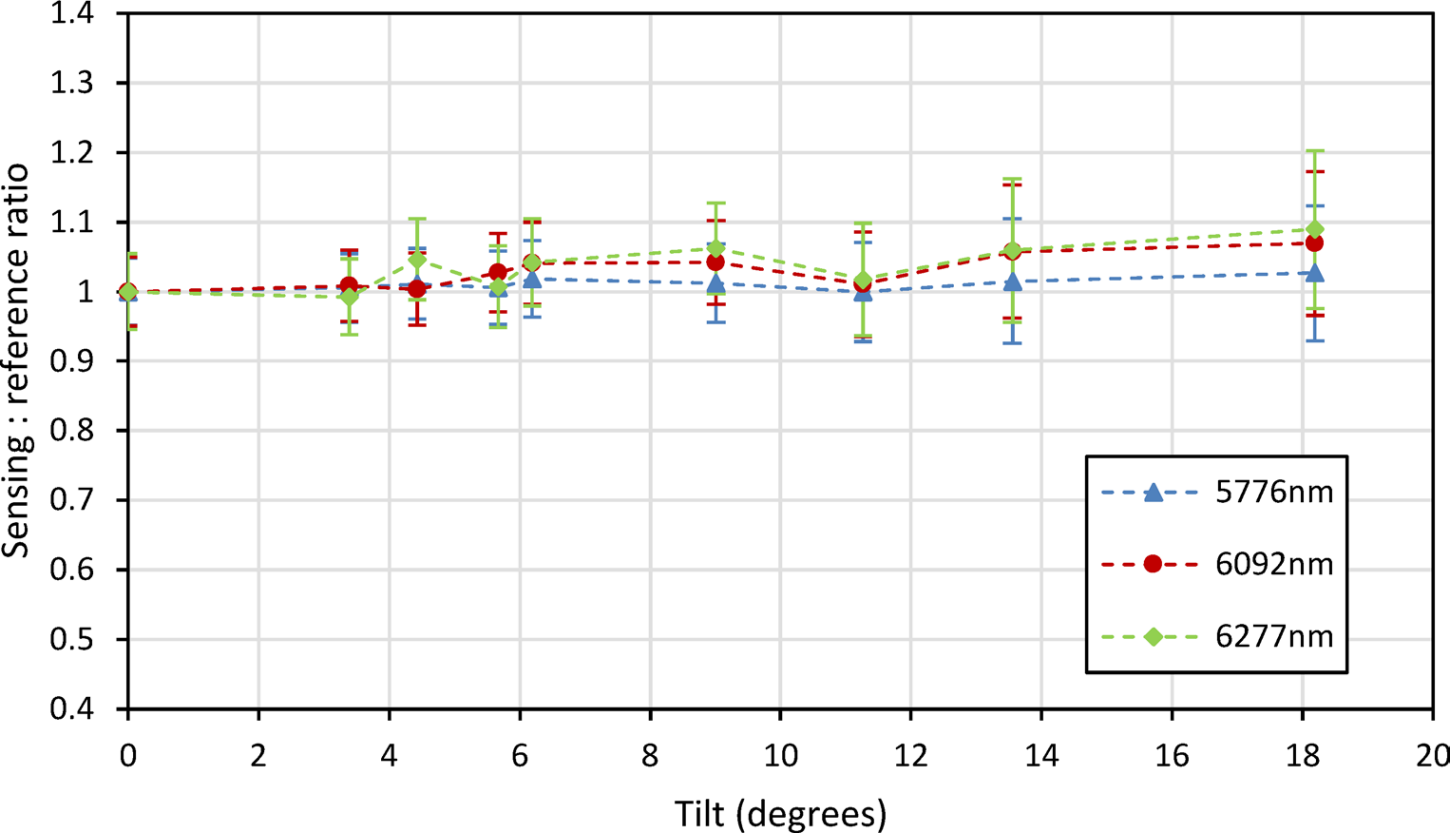
Instruments response to changes in the angle of the sample at the measurement point after being mounted onto the modified trolley.

The first of these, the height measurement as shown in Fig. 19, showed a notable improvement over the previous lab-based setup, with the instrument able to withstand changes in height of at least ± 40 mm. This is within a 10% uncertainty from the zero-height measurement, averaged across the three lasers, with two of these lasers, 6092 nm (1641 cm^−1^) and 6277 nm (1593 cm^−1^), falling significantly below this level. The tilt measurements, shown in Fig. 20, also display an improved result, with the system able to withstand changes in tilt of up to at least 18 degrees (within the same uncertainty window). These improvements are both attributed to improved alignment of the 4 laser beams.

**Fig. 21:**
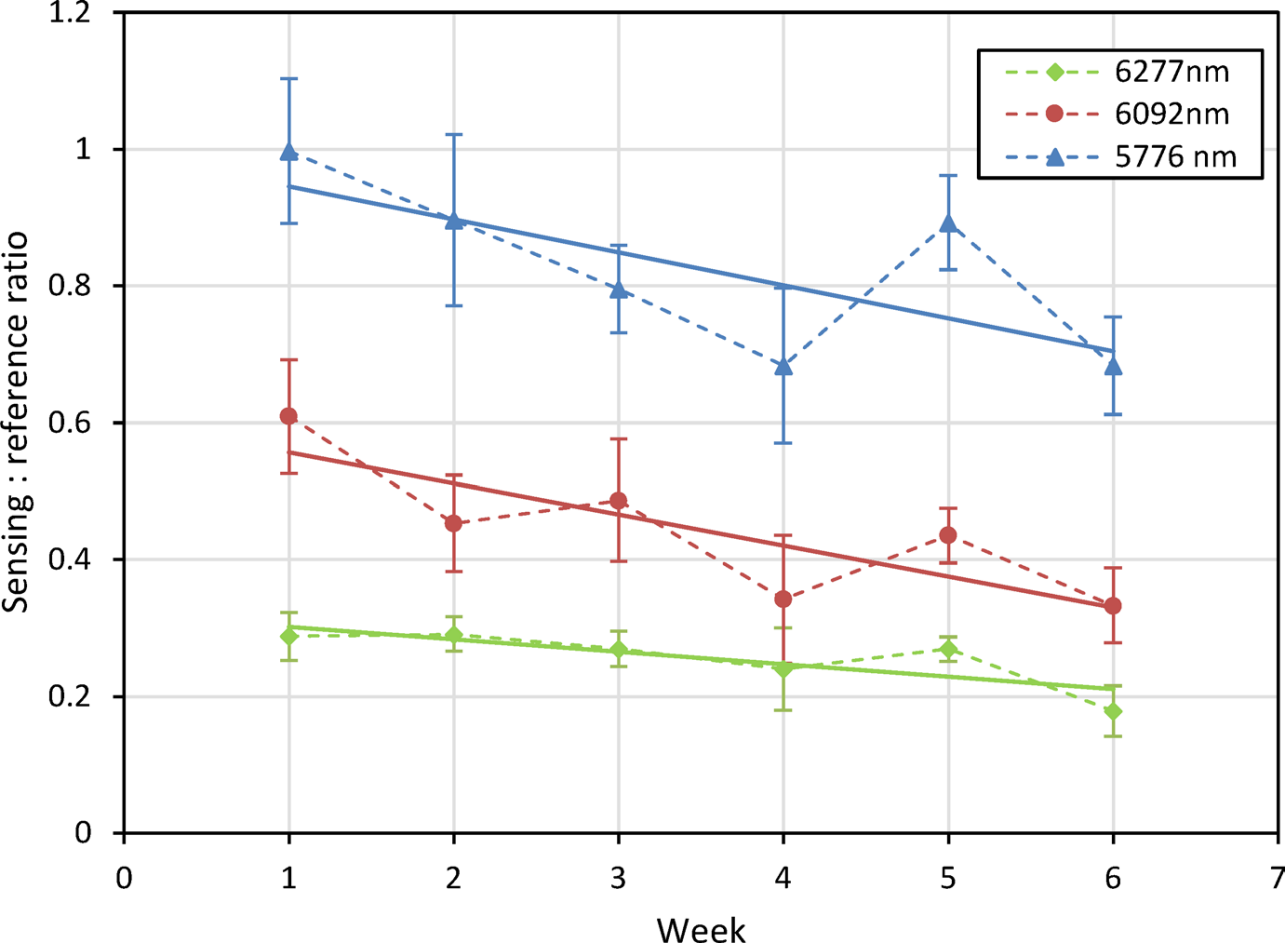
Measurements of signal response of 1–6 week aged bitumen for each referenced QCL after being mounted onto the modified trolley. Solid lines show the trend in signal response for each laser with respect to bitumen age.

**Fig. 22:**
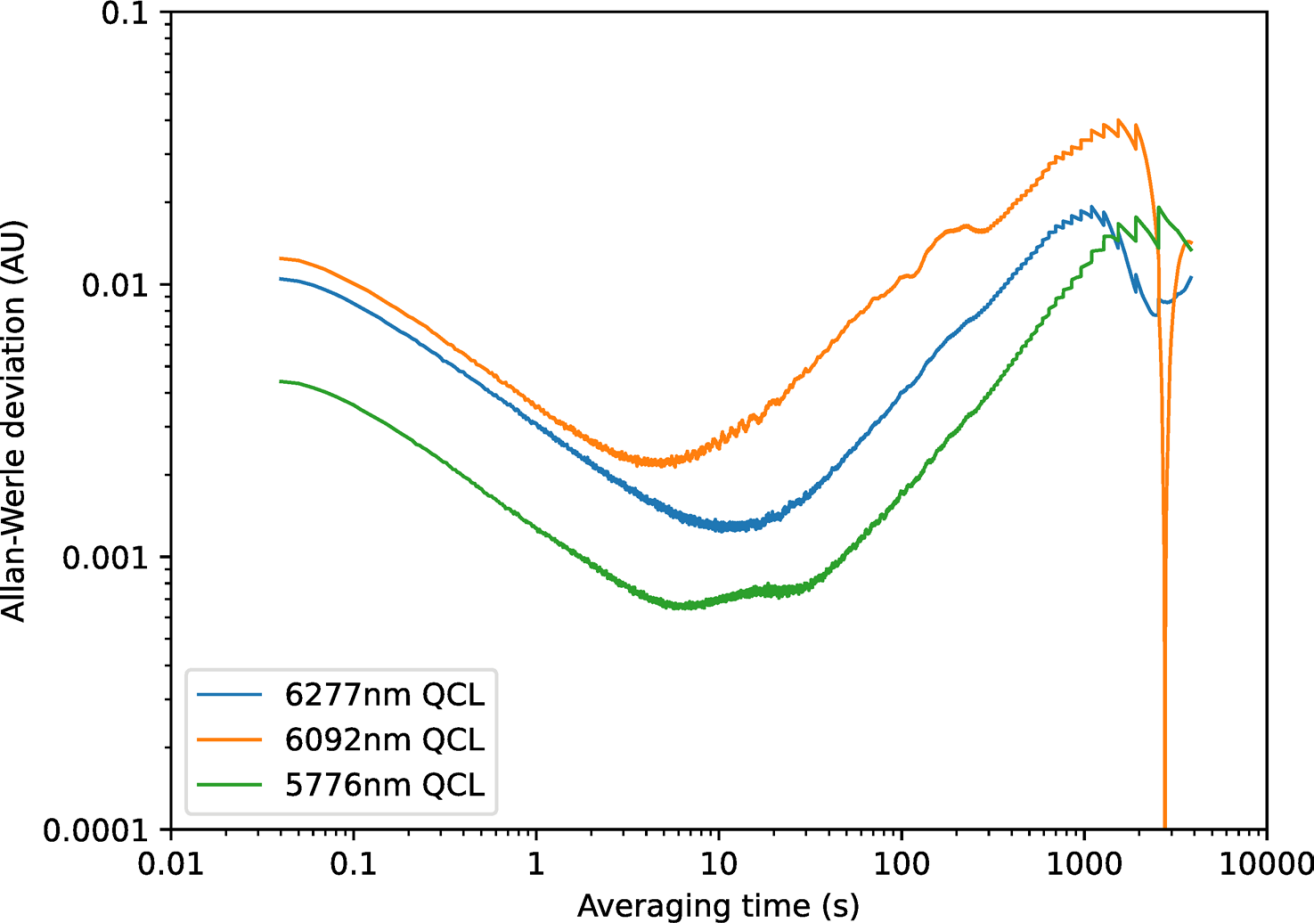
Allan-Werle deviation measurement of 1-week UV-aged bitumen after being mounted onto the modified trolley.

Repeated measurements of 1–6 week aged bitumen, as shown in Fig. 21, displayed a similar trend in signal response with age as previously seen in the lab-based setup. Improvements in the signal response can also be observed in this data, including a more pronounced gradient for each laser, and reduced scatter and error bars. An Allan-Werle deviation measurement was then performed on the sample of 1-week UV-aged bitumen, as shown in Fig. 22. From this, it can be observed that a minimum detectable absorbance below 0.003 AU for all three lasers at averaging times of 2–10 s is possible, with drift reaching a maximum of approximately 0.04 AU.

A preliminary outdoor measurement and shakedown test was undertaken. This was performed to ensure the instrument was operating as expected and that it could survive being exposed to external factors such as vibrations or uneven road surfaces.

**Fig. 23:**
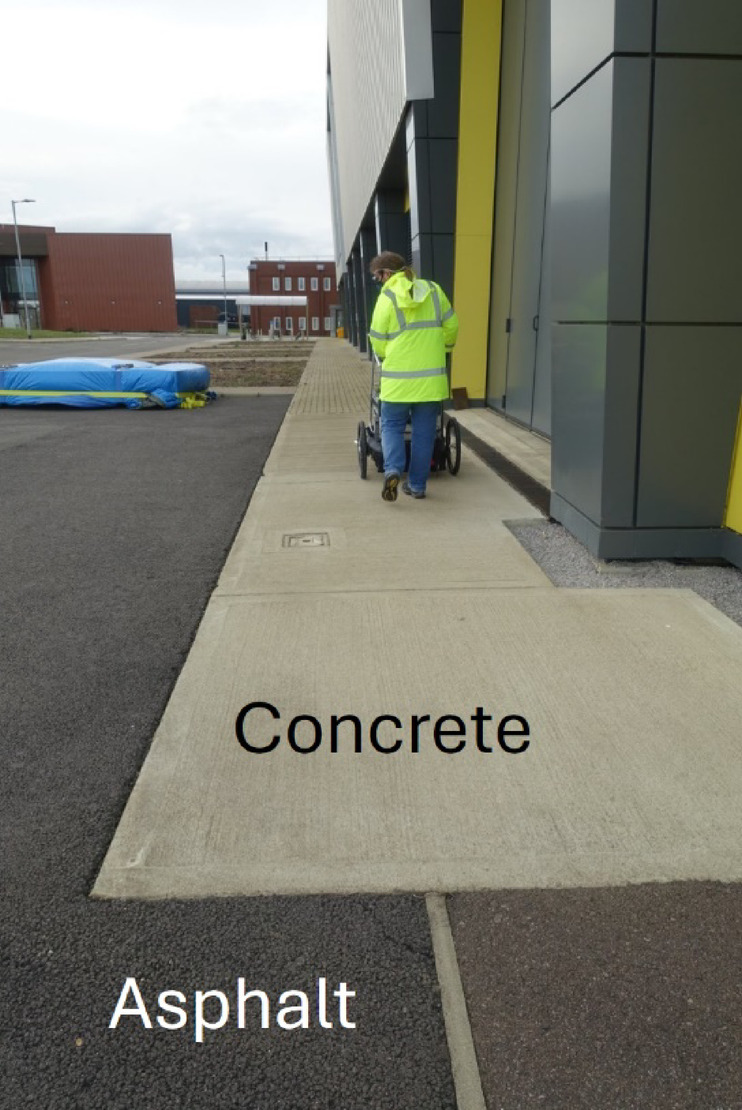
Photograph of location used to make measurements of instrument response to change in surface material (asphalt to concrete).

**Fig. 24:**
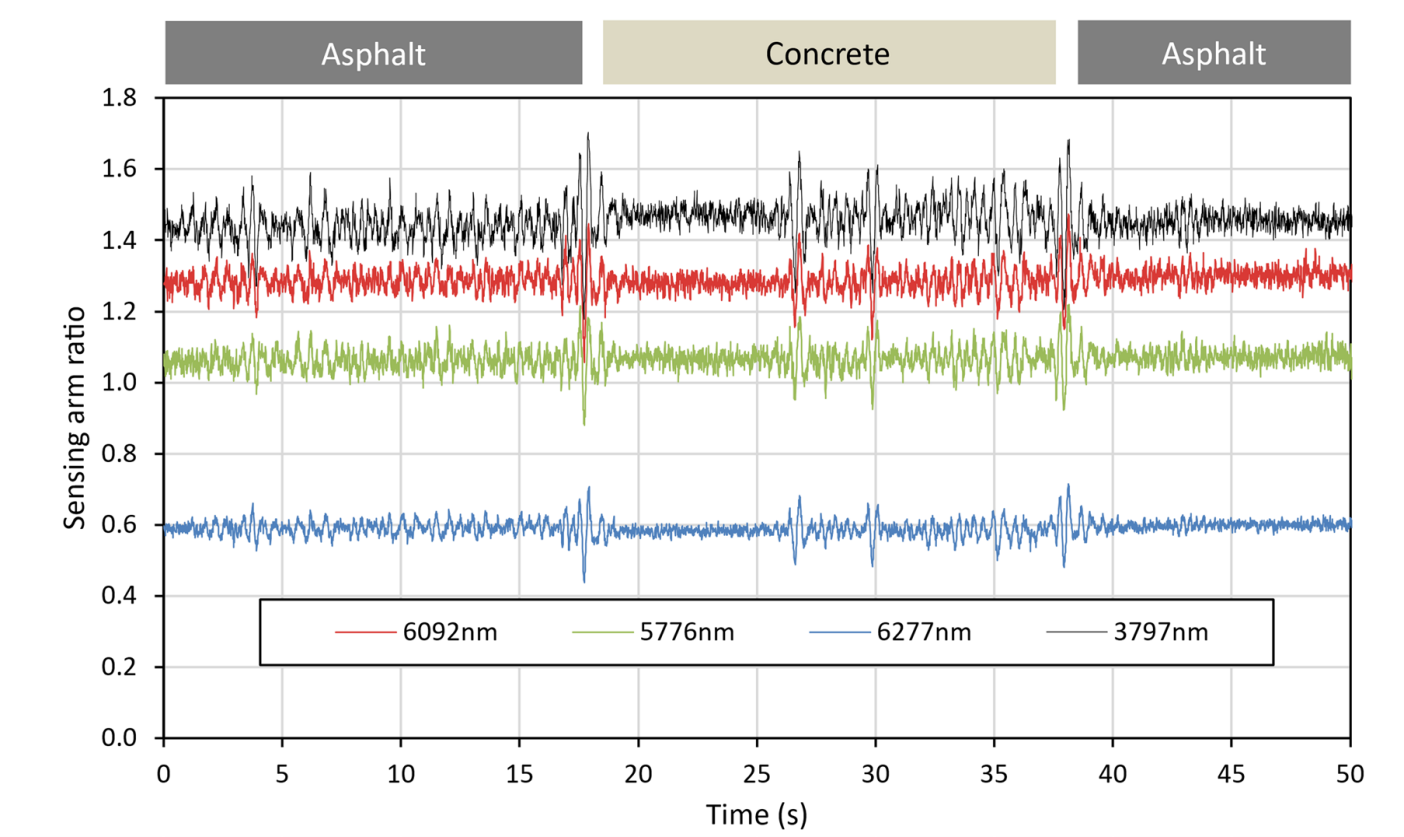
Raw data (ratio of sensing arm to reference arm) for each laser whilst instrument was transitioning between different surface materials.

**Fig. 25:**
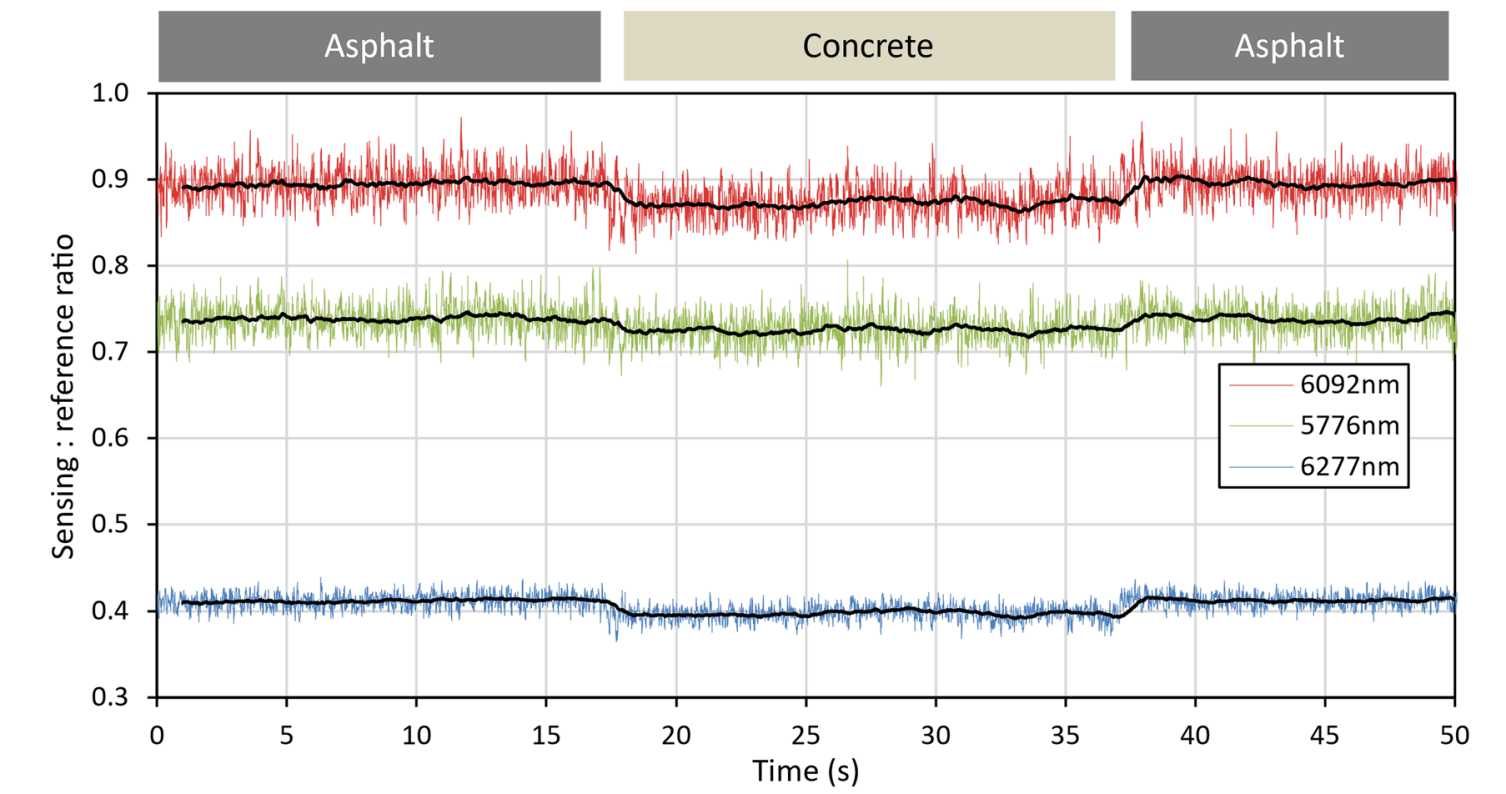
Referenced QCL readings for measurements taken whilst transitioning between different surface materials. Black lines show a 1 s moving average.

To ensure the instrument could detect a change in surface material, the trolley was pushed over a flat surface from asphalt, to concrete, then back to asphalt, as shown in Fig. 23. Although the instrument was not designed to measure concrete, this served as a repeatable test that it could reliably measure a change in reflectivity. The raw data from this measurement run can be observed in Fig. 24, with the referenced data displayed in Fig. 25. As the instrument transitioned between the asphalt and the concrete, as well as between the individual concrete slabs, significant spikes in the raw data can be observed, likely attributable to vibration through the framework of the instrument as the wheels passed over the joins. Once the QCL signals were referenced to the ICL, these spikes in the data cancelled out. Once referenced, the results indicated that a change in surface could be detected. As the trolley moved from asphalt to concrete, a drop in signal strength could be observed, with it returning to its original level when moved back onto the asphalt.

**Fig. 26:**
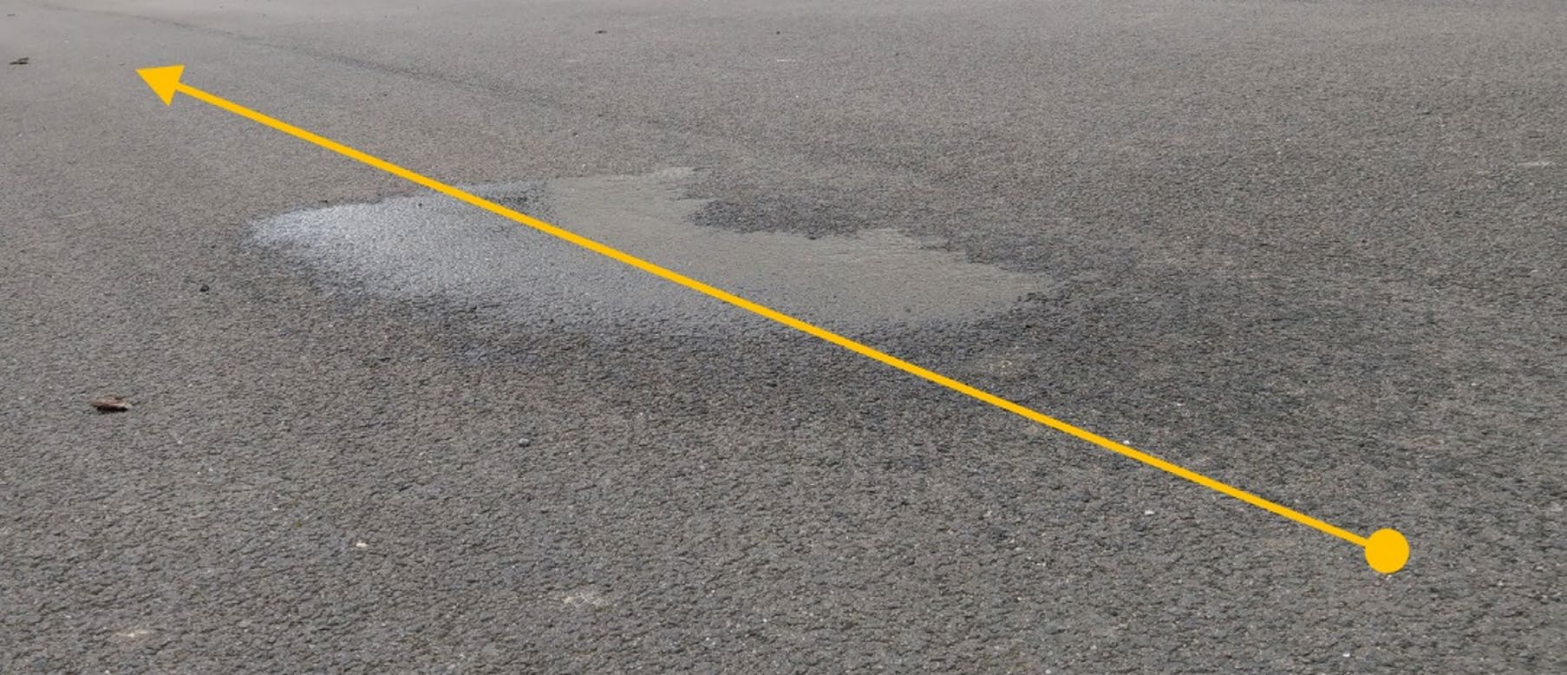
Photograph of wet asphalt used to test instruments response to the presence of water, with arrow showing direction of travel. Regions of dry, damp and wet (shiny) ashpalt can be seen.

**Fig. 27:**
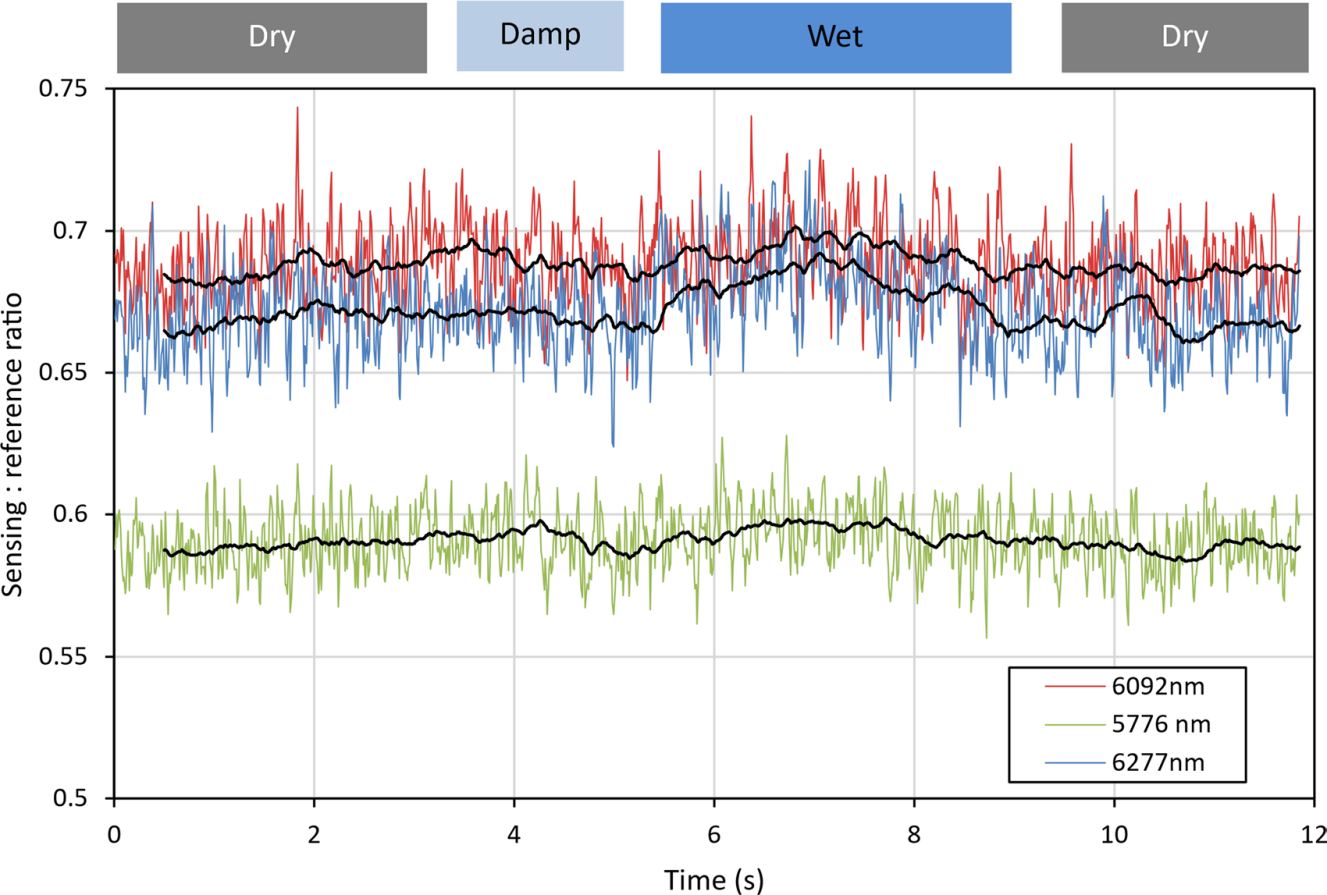
Referenced QCL readings for measurements of response to wet asphalt. Black lines indicate a 0.5 s moving average.

Following this, the instrument was pushed over a small section of wet asphalt, as shown in Fig. 26, in order to determine how it would respond to the presence of liquid water on the road surface. The resultant measurements, as shown in Fig. 27, demonstrate that a small change in the signal strength can be observed as the instrument transitioned from dry asphalt at the start, to wet asphalt around 7 s into the measurement, then back to dry at the end. As mentioned earlier, the instrument was designed to be used in dry conditions only, as is the case for some existing survey instruments. From these results it is clear that the instrument would not be able to operate in wet/damp conditions, thus validating the original design constraints.

## Conclusions

Instrumentation has been developed to measure spectroscopic changes in UV-aged bitumen. This measurement faced two main challenges: optical absorption by water vapour across the region of interest, plus a need to measure reflectivity at a wide range of wavelengths at high speed.

### Key findings

Three discrete lasers operating at wavelengths across the 6 μm (1650 cm^−1^) region were chosen, with a 4th operating at a spectral reference wavelength substantially unaffected by asphalt ageing (3797 nm, 2634 cm^−1^). These lasers had the advantage of narrow linewidths, enabling their use within narrow water absorption windows, and rapid modulation via the injection current, enabling measurement of reflectivity quasi-simultaneously across all 4 wavelengths (by which it is meant that the modulation periods were > 10 × smaller than the distance scale of asphalt granularity at a travel speed of 20 m s^−1^). Use of the spectral reference was shown in testing to provide compensation for bulk changes in optical throughput caused, for example, by changes in surface height and tilt, or by levels of dirt on the optics closest to the exposed beam. The reference wavelength compensated for the effects of vibration caused by a change of surface height during outdoor tests.

Water vapour was particularly troublesome because its concentration in the air surrounding the instrument was expected to be highly variable over short timescales as a result of vehicle exhaust emission. Dry air purging, as used for FTIR measurement, would be problematic for a portable instrument and almost impossible to achieve well for the free section of optical path spanning the standoff distance.

The emission wavelength of the lasers were chosen to sit within known transmission windows between water absorption lines. This reduced the unwanted absorption to a manageable but non-zero residual level. To compensate for the expected variability in absorption, a pathlength-matched reference beam was also created that did not extend to the asphalt surface but instead was reflected by a mirror into a second detector. It was intended that this beam would experience the same level of water vapour as the measurement beam, to provide automatic compensation for the resultant changes in recorded light intensity. For laboratory and outdoor measurements, this compensation was effective.

The best standoff geometry was a compromise between the consequences of parallax and the desire to suppress specular reflections. The final geometry used a 15° incident beam and normal collection axis. The instrument was mounted onto a modified trolley for outdoor testing. To determine the instruments orientation tolerance, measurements of height and tilt sensitivity were performed, with a demonstrated ± 40 mm tolerance for changes in height and a 18° tolerance for tilt within a 10% uncertainty averaged across the three lasers. Measurements of bitumen subjected to an artificial, accelerated ageing process showed a clear trend being observed between 1 and 6 week aged samples, with the signal-to-reference ratio decreasing as the bitumen aged. The percentage change in signal intensity observed for these measurements is significantly larger than the variation observed in the height and tilt measurements, especially after being mounted onto the trailer, indicating that the change seen is of significance.

Outdoor test results indicated the instrument could detect changes in surface material, and confirming that the spectral refencing compensated for the effects of bumps. These tests also confirmed that liquid water on the surface of the asphalt would cause spurious readings; this is not anticipated to be a problem as some existing survey tools also require operation in dry conditions. Although undertaken at walking speed, these measurements are believed to be transferable and can be directly related to ones taken at traffic speed.

The use of 4 discrete laser sources was itself problematic. The 4 beams were difficult to align, though once aligned they remained so during testing, including outdoor tests. Alignment generally improved throughout the course of the development. Parallax affected not only the registration between emitted and collected beams but also (to a lesser extent) the registration between the 4 different wavelengths.

### Future scope

Measurement at only 3 wavelengths across this region could make spectra hard to interpret in comparison with the continuous spectra obtained in FTIR studies^7^. In future, use of mid-IR optical fibre couplers in the beam delivery section of the optics might help with beam registration of multiple lasers. These were unavailable at the start of this work but a commercial option is now available, albeit multimode^28^. Alternative sources would also be of interest. QCL arrays are now available with 32 individually addressable, co-aligned pulsed lasers^29^; this would help with both beam registration and in providing a quasi-continuous reflectance spectrum (the target spectral features are broad and do not in themselves require narrow resolution measurement). This technology has been used as the basis for a portable spectrometer for standoff measurement of diffuse backscatter from residues of hazardous materials^30^. However, as the individual lasers are pulsed, the resulting emission linewidths may prove troublesome within the water windows, and individual tuning of each laser’s emission would be needed to locate it within each window.

The development of fast spectrometers in the 5–7 μm region would be beneficial. Grating spectrometers are now available at low cost in the 2–5 μm and 7.6–12 μm regions, offering high-speed and sensitive spectral measurement by making use of wavelength upconversion^31,32^. They could be paired with a supercontinuum source with high spectral brightness. However, resolution is currently too broad to permit operation within spectral windows (and fundamentally limited by the upconversion process). Alternatively the development of dual comb spectrometers in the mid-IR^33^ might enable full spectral measurement at high speed and with suitably narrow emission linewidth so that good measurements can be made within each window.

### Overall summary

In summary, it has been demonstrated that fast acquisition rate measurements of surface reflectance have been made at walking speed and within a spectral region that is often avoided because of the ubiquitous presence of water vapour. This work could open up this region to measurement in other applications such as those that have adopted near IR reflectance, for example in art, polymer identification and processing, and pharmaceuticals—any application where the target material is dry. It is hoped that the success of this work will also foster the development of new sources, spectrometers and transmitting optics, since this region is sparsely served by available technology.

Finally, this work takes measurements a step closer to the quantification of chemical ageing of asphalt on in-service roads. By using the same underlying spectroscopy in the measurement, it could provide a useful scientific link between well-established laboratory measurements of ageing and real-world failure mechanisms. Work will be needed to establish the precise meaning of spectroscopic readings for operators, which might require substantial efforts to characterise different types of asphalt and establish thresholds at which preventative maintenance is required. It is believed that measurements of reflectance in the C=O region could provide an important (and currently missing) piece of the overall puzzle of in-service asphalt ageing, alongside use of more well-established characterisation methods.

## Data Availability

Data supporting this study are openly available from the Cranfield Online Research Database (CORD), at 10.57996/cran.ceres-2674.

## References

[1] Wright, A. et al. Hi-SPEQ—developing the technical and quality requirements for high-speed condition surveys of road networks. *Transport. Res. Proc.* **14**, 2956–2965 (2016).

[2] Pynn, J., Wright, A. & Lodge, R. Automatic identification of cracks in road surfaces. In *7*^*th*^* International Conference on Image Processing and its Applications*. 671–675 (1999).

[3] Abouelsaad, A. & White, G. Review of asphalt mixture ravelling mechanisms, causes and testing. *Int. J. Pavement Res. Technol.* **15**(6), 1448–1462 (2022).

[4] McRobbie, S., Iaquinta, J., Wright, A., Trumper, P. & Kennedy, J. Development and validation of algorithms for the automatic detection of fretting based on multiple line texture data. Research into pavement surface disintegration: Phase 2—interim report. *TRL Published Project Report* **PPR628**, (Transport Research Laboratory, 2012).

[5] Hofko, B., Maschauer, D., Steiner, D., Mirwald, J. & Grothe, H. Bitumen Ageing—Impact of reactive oxygen species. *Case Stud. Const. Mater.* **13**, e00390 (2020).

[6] Nivitha, M. R., Prasad, E. & Krishnan, J. M. Ageing in modified bitumen using FTIR spectroscopy. *Int. J. Pavement Eng.* **17**, 565–577 (2016).

[7] Hou, X. D., Lv, S. T., Chen, Z. & Xiao, F. P. Applications of Fourier transform infrared spectroscopy technologies on asphalt materials. *Measurement* **121**, 304–316 (2018).

[8] Bowden, H., Almond, M. J., Hayes, W., Browne, C. & McRobbie, S. The use of diffuse reflectance infrared spectroscopy to monitor the oxidation of UV irradiated and naturally aged bitumen and asphalt. *Road Mater. Pavement Des.* **22**, 1254–1267 (2019).

[9] Neo, E. R. K., Yeo, Z., Low, J. S. C., Goodship, V. & Debattista, K. A review on chemometric techniques with infrared, Raman and laser-induced breakdown spectroscopy for sorting plastic waste in the recycling industry. *Resour. Conserv. Recy.* **180**, 106217 (2022).

[10] Okano, M. & Watanabe, S. Inspection of internal filler alignment in visibly opaque carbon-black–rubber composites by terahertz polarization spectroscopy in reflection mode. *Polym. Test.* **72**, 196–201 (2018).

[11] Wallin, S., Pettersson, A., Östmark, H. & Hobro, A. Laser-based standoff detection of explosives: A critical review. *Anal. Bioanal. Chem.* **395**, 259–274 (2009). 19484226 10.1007/s00216-009-2844-3

[12] Woodcock, T., Downey, G. & O’Donnell, C. P. Better quality food and beverages: The role of near infrared spectroscopy. *J. Near Infrared Spec.* **16**(1), 1–29 (2008).

[13] Mendez, J., Mendoza, L., Cruz-Tirado, J. P., Quevedo, R. & Siche, R. Trends in application of NIR and hyperspectral imaging for food authentication. *Sci. Agropecuaria* **10**(1), 143–161 (2019).

[14] Ollinger, S. V. Sources of variability in canopy reflectance and the convergent properties of plants. *New Phytol.* **189**(2), 375–394 (2011). 21083563 10.1111/j.1469-8137.2010.03536.x

[15] Cozzolino, D., Porker, K. & Laws, M. An overview on the use of infrared sensors for in field, proximal and at harvest monitoring of cereal crops. *Agriculture-Basel* **5**(3), 713–722 (2015).

[16] Tsuchikawa, S., Ma, T. & Inagaki, T. Application of near-infrared spectroscopy to agriculture and forestry. *Anal. Sci.* **38**(4), 635–642 (2022). 35347675 10.1007/s44211-022-00106-6

[17] Angelopoulou, T., Tziolas, N., Balafoutis, A., Zalidis, G. & Bochtis, D. Remote sensing techniques for soil organic carbon estimation: A review. *Remote Sens.* **11**(6), 676 (2019).

[18] Daffara, C., Pampaloni, E., Pezzati, L., Barucci, M. & Fontana, R. Scanning multispectral IR reflectography SMIRR: An advanced tool for art diagnostics. *Accounts Chem. Res.* **43**(6), 847–856 (2010). 10.1021/ar900268t 20230039

[19] Cucci, C., Delaney, J. K. & Picollo, M. Reflectance hyperspectral imaging for investigation of works of art: Old master paintings and illuminated manuscripts. *Accounts Chem. Res.* **49**(10), 2070–2079 (2016). 10.1021/acs.accounts.6b00048 27677864

[20] Galán-Freyle, N., Pacheco-London, L. C., Román-Ospino, A. D. & Hernandez-Rivera, S. P. Applications of quantum cascade laser spectroscopy in the analysis of pharmaceutical formulations. *Appl. Spectrosc.* **70**(9), 1511–1519 (2016). 27558366 10.1177/0003702816662609

[21] Breshike, C. J. et al. Infrared backscatter imaging spectroscopy of trace analytes at standoff. *J. Appl. Phys.* **125**, 104901 (2019).

[22] Hensley, J. M. et al. Standoff detection from diffusely scattering surfaces using dual quantum cascade laser comb spectroscopy. *Proc. SPIE* **10638**, 1063820 (2018).

[23] Hodgkinson, J. et al. Mid-IR standoff measurement of ageing-related spectroscopic changes in bitumen in the 6 µm (1700 cm^−1^) region. Part 1: Measurement strategy and instrument design principles. *Sci. Rep.* **15**, 25041 (2025). 10.1038/s41598-025-01135-7 PMC12254496 40645953

[24] Saleh, N. F. et al. Field calibration and validation of a pavement aging model. *Int. J. Pavement Eng.* **24**(2), 2027416 (2022).

[25] Bowden, H. *Spectroscopic Analysis of Roads at Traffic Speed*. PhD Thesis, University of Reading, Reading (2018).

[26] A Guide to Reflectance Coatings and Materials. Tech Guide, Labsphere, North Sutton, NH, USA. Available at https://pro-lite.co.uk/wp-content/uploads/2022/03/Labsphere-Technical-Guide-Coatings-Materials.pdf [accessed 27/10/23]

[27] Werle, P., Mücke, R. & Slemr, F. The limits of signal averaging in atmospheric trace-gas monitoring by tunable diode-laser absorption spectroscopy (TDLAS). *Appl. Phys. B* **57**(2), 131–139 (1993).

[28] IR Flex Product Datasheet. Fused Fiber Combiner: Chalcogenide MWIR Fused Fiber Combiner (1.5 to 6.5µm). (IR Flex, 2022). Available at: www.irflex.com [Accessed 22/6/23]

[29] Lewicki, R., Witinski, M., Li, B. & Wysocki, G. Spectroscopic benzene detection using a broadband, monolithic DFB-QCL array. *Proc. SPIE* **9767**, 97671T (2016).

[30] Witinski, M. et al. Portable standoff spectrometer for hazard identification using integrated quantum cascade laser arrays from 6.5 to 11 μm. *Opt. Express* **26**(9), 12159–12168 (2018). 29716130

[31] Friis, S. M. M. & Høgstedt, L. Upconversion-based mid-infrared spectrometer using intra-cavity LiNbO3 crystals with chirped poling structure. *Opt. Lett.* **44**(17), 4231–4234 (2019). 31465369 10.1364/OL.44.004231

[32] Rodrigo, P. J. et al. Room-temperature, high-SNR upconversion spectrometer in the 6–12 µm region. *Laser Photon. Rev.* **15**(3), 2000443 (2021).

[33] Liao, R. et al. Dual-comb generation from a single laser source: Principles and spectroscopic applications towards mid-IR—A review. *J. Phys. Photonics* **2**, 042006 (2020).

